# Perinatal depression and risk of mortality: nationwide, register based study in Sweden

**DOI:** 10.1136/bmj-2023-075462

**Published:** 2024-01-10

**Authors:** Naela Hagatulah, Emma Bränn, Anna Sara Oberg, Unnur A Valdimarsdóttir, Qing Shen, Donghao Lu

**Affiliations:** 1 Clinical Research Center for Mental Disorders, Shanghai Pudong New Area Mental Health Center, Tongji University School of Medicine, Shanghai, China; 2 Institute for Advanced Study, Tongji University, Shanghai, China; 3 Unit of Integrative Epidemiology, Institute of Environmental Medicine, Karolinska Institutet, Stockholm, Sweden; 4 Department of Epidemiology, Harvard TH Chan School of Public Health, Boston, MA, USA; 5 Department of Medical Epidemiology and Biostatistics, Karolinska Institutet, Stockholm, Sweden; 6 Centre of Public Health Sciences, Faculty of Medicine, University of Iceland, Reykjavík, Iceland

## Abstract

**Objective:**

To determine whether women with perinatal depression are at an increased risk of death compared with women who did not develop the disorder, and compared with full sisters.

**Design:**

Nationwide, register based study.

**Setting:**

Swedish national registers, 1 January 2001 to 31 December 2018.

**Participants:**

86 551 women with a first ever diagnosis of perinatal depression ascertained through specialised care and use of antidepressants, and 865 510 women who did not have perinatal depression were identified and matched based on age and calendar year at delivery. To address familial confounding factors, comparisons were made between 270 586 full sisters (women with perinatal depression (n=24 473) and full sisters who did not have this disorder (n=246 113)), who gave at least one singleton birth during the study period.

**Main outcome measures:**

Primary outcome was death due to any cause. Secondary outcome was cause specific deaths (ie, unnatural and natural causes). Multivariable Cox regression was used to estimate hazard ratios of mortality comparing women with perinatal depression to unaffected women and sisters, taking into account several confounders. The temporal patterns of perinatal depression and differences between antepartum and postpartum onset of perinatal depression were also studied.

**Results:**

522 deaths (0.82 per 1000 person years) were reported among women with perinatal depression diagnosed at a median age of 31.0 years (interquartile range 27.0 to 35.0) over up to 18 years of follow-up. Compared with women who did not have perinatal depression, women with perinatal depression were associated with an increased risk of death (adjusted hazard ratio 2.11 (95% confidence interval 1.86 to 2.40)); similar associations were reported among women who had and did not have pre-existing psychiatric disorder. Risk of death seemed to be increased for postpartum than for antepartum depression (hazard ratio 2.71 (95% confidence interval 2.26 to 3.26) *v* 1.62 (1.34 to 1.94)). A similar association was noted for perinatal depression in the sibling comparison (2.12 (1.16 to 3.88)). The association was most pronounced within the first year after perinatal depression but remained up to 18 years after start of follow up. An increased risk was associated with both unnatural and natural causes of death among women with perinatal depression (4.28 (3.44 to 5.32) *v* (1.38 (1.16 to 1.64)), with the strongest association noted for suicide (6.34 (4.62 to 8.71)), although suicide was rare (0.23 per 1000 person years).

**Conclusions:**

Even when accounting for familial factors, women with clinically diagnosed perinatal depression were associated with an increased risk of death, particularly during the first year after diagnosis and because of suicide. Women who are affected, their families, and health professionals should be aware of these severe health hazards after perinatal depression.

## Introduction

Perinatal depression is defined as depression that occurs during pregnancy (antepartum depression) or weeks after delivery (postpartum depression), affecting about 10-20% of women.^1^ ^2^ Perinatal depression can be diagnosed using the Diagnostic and Statistical Manual of mental disorders, fourth edition criteria from 1994, or using the updated fifth edition from 2013 and onward.^3^ Occurring during a vulnerable period in life, perinatal depression can have a detrimental effect on maternal wellbeing.^4^ For instance, perinatal depression has been linked to an increased risk of suicide,^4^ which accounts for up to 20% of maternal deaths postpartum in high income countries.^5^ Nevertheless, little is known about the overall increased mortality in women with perinatal depression.

Studies have found that women with postpartum psychiatric disorders, including psychotic, affective, and anxiety disorders, have an increased risk of death compared with either women who were not affected^6^ or with the general female population.^7^ ^8^ However, the association between perinatal depression, one of the most common complications of pregnancy,^9^ and risk of mortality among affected women, has not been examined specifically. Prior studies have considered only postpartum onset disorders,^6^ ^7^ ^8^ and indications show that antepartum and postpartum onset of depression differ in prevalence,^2^ symptom severity,^10^ and suicidal behaviour.^5^ ^11^ Furthermore, familial factors, such as genetic factors and shared childhood experiences, may contribute to the risks of perinatal depression 12 13 and mortality due to suicide;^14^ ^15^ yet, no study to date has addressed these issues.

We aimed to investigate the association of perinatal depression attended by specialised care or treated by antidepressants, with subsequent risk of death. We hypothesised that perinatal depression is associated with an increased risk of mortality.

## Methods

### Study design

We conducted a matched cohort study based on the nationwide population, followed by a sibling comparison to further control for shared familial confounding, by leveraging the nationwide health registers in Sweden. The medical birth register covers almost all births in Sweden since 1973.^16^ The national patient register has almost complete coverage on all inpatient care since 1987 and outpatient specialised care from 2001 onwards.^17^ The Swedish national drug register contains information about dispensed prescribed medications from all pharmacies in Sweden since July 2005.^18^ The causes of death register covers a complete record of all deaths in Sweden since 1952.^19^ We used the personal identification number uniquely assigned to each resident for linkage cross registers. 

We identified all pregnancies in the medical birth register between 1 January 2001 and 31 December 2017 (n=1 803 987; 1 041 419 women). We excluded multiple gestations (n=51 806), subsequent pregnancies due to an incident perinatal depression (n=35 133), or pregnancies with incomplete or erroneous records (n=681), leaving 1 716 367 pregnancies from 1 029 215 women in the study base (supplementary fig S1). We identified 86 551 women who received a first ever diagnosis of perinatal depression (ie, without prior depression diagnosis during pregnancy or one year postpartum) attended by specialised care or treated by antidepressants, including antepartum or postpartum depression. We used the term women to be in alignment with previous literature, although individuals included in this study may identify differently. For each women, we randomly selected 10 women who did not have this disorder from the study base, and matched them using an incidence density sampling method.^20^ Women who did not have perinatal depression at the same gestational or postpartum day when the index woman received her perinatal depression diagnosis (n=865 510; the reference group) were matched on the basis of their age (plus or minus 1 year) and same calendar year at delivery; date of perinatal depression diagnosis or matching was used as the index date. Women were followed up from the index date or from the delivery date, whichever came later, until emigration (as designated from the migration register), death, a diagnosis of perinatal depression (in reference group), or 31 December 2018, whichever occurred first (supplementary fig s2).

For the sibling comparison analysis, we used the Swedish multigenerational register, which includes information about parents for Swedish residents born since 1932.^21^ We included all women with at least one full sister and who give live birth during the study period (ie, not only families that have sisters affected by perinatal depression). We followed up all full sisters from the date of their first delivery until emigration, death, or 31 December 2018, whichever came first (supplementary fig S2). 

### Ascertainment of perinatal depression

Perinatal depression was defined as any diagnosis of depression through specialised care or dispensation of antidepressants during pregnancy and up to one year after delivery,^22^ recorded in the medical birth register, national patient register, or prescribed drug register. The gestational age was estimated from the routine ultrasound examination offered to all pregnant women.^23^ Start of pregnancy was defined as the date obtained from subtracting the estimated gestational age from the delivery date. We considered both primary and secondary diagnoses to identify perinatal depression cases, using the 10th Swedish revision of the International Classification of Diseases codes (supplementary table S1). Although a validity study has not been carried out specifically for perinatal depression, the diagnosis of depression has been validated in the national patient register.^24^ We also identified use of antidepressant medication in the medical birth register and dispensations in the prescribed drug register using the anatomical therapeutic chemical code as a proxy for perinatal depression. The incident date of perinatal depression was defined as the date of the first recorded diagnosis or the prescribing date of a filled antidepressant, whichever came first. Women meeting our definition of perinatal depression were categorised into antepartum depression (if the date of perinatal depression occurred during pregnancy) and postpartum depression (if no perinatal depression was identified during pregnancy but was identified within one year after delivery; supplementary table S1).

### Ascertainment of outcome

The key outcome of interest was deaths due to any cause. We ascertained all causes of deaths from the causes of death register, and based on the underlying cause, categorised the data into death due to unnatural (ie, external causes) or natural causes (ie, disease related causes) (supplementary table S1). Additionally, we investigated the most common underlying causes of death in this population, that is, due to suicide, accident, cancer, and cardiovascular disease. Reporting of deaths is mandatory in Sweden with more than 96% of deaths with causes recorded.^19^ The accuracy of the causes of death has been found high, particularly for deaths due to cancer, cardiovascular disease, and unnatural causes.^19^ Mortality was determined by death rate, from the index date until the end of follow-up.

### Covariates

We obtained information about women’s demographics from the medical birth register and socioeconomic status from the Longitudinal Integration Database for Health Insurance and Labour Market, as known risk factors for both depression and premature death.^25^ ^26^ Some characteristics specific to pregnancy, and pre-existing psychiatric disorders (any time before pregnancy) are putative risk factors for both perinatal depression and mortality,^27^ ^28^ ^29^ and were extracted from the medical birth register and national patient register. Adverse birth outcomes could affect the risk of postpartum depression^30^ and maternal death,^31^ hence, information about mode of delivery, gestational age, birth weight, and stillbirth was obtained from the medical birth register. Death of a child within the first year after birth was identified through the causes of death register (supplementary table s1).

### Statistical analysis

Crude mortality rate was assessed by dividing the total number of deaths by the accumulated person years at risk. We assessed the association between perinatal depression and mortality by calculating hazard ratios and their 95% confidence intervals from Cox regression models stratified by risk set (individually matched on age and calendar year at delivery), using time since the index date as the underlying time scale. The proportional hazard assumption was tested by use of the Schoenfield residual plot and was held in both population and sibling analyses. The association was examined for women with perinatal depression overall and separately for women who either had or did not have a pre-existing psychiatric disorder. To show the effect by various source of confounding, we assessed the following three models in a stepwise manner: 1) crude; 2) adjusted for sociodemographic factors (ie, educational level, household income, country of birth, and cohabitation status); 3) additionally adjusted for pregnancy specific characteristics (smoking and body mass index during early pregnancy, parity, pre-existing psychiatric disorder (overall association), and diabetic and hypertensive disorders in pregnancy). Model 3 was interpreted as the main model. For all covariates, missing value was grouped as level “unknown.” We performed a complete case analysis that included women with no missing value on any covariate. We also assessed the association separately for antepartum and postpartum depression. Because birth outcomes (ie, mode of delivery, birth weight, gestational age, and death of child) are mediators for the association between antepartum depression and maternal death (supplementary figure S3), we only adjusted for birth outcomes for postpartum depression. In the sibling analysis, we compared the risk between full sisters, using Cox regression models stratified by family identifier as risk set, using attained age as the underlying time scale. Only sisters discordant on perinatal depression can contribute to the contrast of interest, whereas other sister sets contribute to outcome prediction and covariate effects. In a sensitivity analysis, we considered women with perinatal depression identified from clinical diagnosis or from dispensed medication separately, to assess the effect of different identification sources.

We calculated cumulative all cause mortality rate, as well as cause specific mortality rate, for women who had and did not have perinatal depression, to visualise the change in mortality rates over time. To further visualise temporal patterns, we used flexible parametric survival models to estimate the time varying association between perinatal depression and mortality.^32^ The models were adjusted for matching variables (maternal age and calendar year at delivery) and the aforementioned covariates. To provide readouts for clinicians, we derived period specific hazard ratios by years since the index date of perinatal depression diagnosis of one year or less, two to four years, five to nine years, ten years and more, using stratified Cox regression.

To understand the driving forces underlying increased risk of mortality, we estimated hazard ratios of cause specific mortality and generated a histogram of the cause specific frequencies over time. Cancer-specific and cardiovascular mortality risks were evaluated among women who had or did not have a prior diagnosis of these respective diseases (any time before index date). We additionally adjusted the association for pre-existing comorbidities, using Charlson Comorbidity Index (any time before index date), to control for the effect of somatic comorbidities.^33^ Additionally, we estimated the association by time since pregnancy for antepartum depression (within 13 weeks or 14 weeks onwards) and by time since delivery for postpartum depression (within six months or 7-12 months). To examine potential effect measure modification, we stratified analyses by age and calendar year at delivery using a cutoff of 2010 when screening for perinatal depression was introduced in national guidelines.

We used SAS 9.4 (SAS Institute) and STATA 17 (StataCorp LP) for all analyses.

### Patient and public involvement

No patients were involved in setting the research question or the outcome measures, nor were they involved in the design, conduct or interpretation of the study. Dissemination to the Swedish population (which constitutes the study population) will be through media outreach (eg, press release and communication) on publication of this study.

## Results

Among 86 551 women with perinatal depression, 55% had antepartum and 45% postpartum depression. The median age at diagnosis was 31.0 years (interquartile range 27.0-35.0) (table 1). Compared with women who did not have perinatal depression, women with perinatal depression were more likely to be born in the Nordic countries, have a lower educational level and household income, and less likely to live with the father of the child. They were more likely to be primiparous and overweight or obese at enrolment to antenatal care, to smoke tobacco, and to have a pre-existing psychiatric disorder. They were also more likely to have undergone a caesarean section and had a death of a child at birth or within the first year (table 1). Similar patterns were noted in the sibling cohort (supplementary table S2). Among all women in the study base with identifiable parents (n=821 540, 79.8%), 28.3% (n=24 473) of the 86 551 women who had perinatal depression had at least one full sister who gave at least one singleton birth during the study period. Of 270 586 sisters, 24 473 women had perinatal depression and 246 113 women did not have this disorder. 

**Table 1:** Baseline demographic characteristics of women with and without perinatal depression in the population based matched cohort. Data are number of women (percentage)

Characteristics	No perinatal depression (n=865 510)	Perinatal depression (n=86 551)
**Demographic characteristics**		
Age at delivery, years:		
≤20	23 197 (2.7)	2309 (2.7)
21-25	136 241 (15.7)	13 687 (15.8)
26-30	261 016 (30.2)	26 032 (30.1)
31-35	268 111 (31.0)	26 760 (30.9)
36-40	144 412 (16.7)	14 480 (16.7)
41-45	31 198 (3.6)	3166 (3.7)
≥46	1335 (0.2)	117 (0.1)
Maternal year of birth, years:		
≤1964	11 059 (1.3)	1094 (1.3)
1965-69	50 531 (5.8)	5071 (5.9)
1970-74	133 269 (15.4)	13 346 (15.4)
1975-79	203 856 (23.6)	20 345 (23.5)
1980-84	219 358 (25.3)	21 943 (25.4)
1985-89	167 935 (19.4)	16 855 (19.5)
1990-94	70 497 (8.1)	6979 (8.1)
≥1995	9005 (1.0)	918 (1.1)
Country of birth*:		
Nordic	662 393 (76.5)	74 499 (86.1)
Other/unknown	203 117 (23.5)	12 052 (13.9)
Educational level, years:		
≤9	103 395 (11.9)	13 924 (16.1)
10-12	324 665 (37.5)	36 211 (41.8)
>12	419 393 (48.5)	35 701 (41.2)
Unknown	18 057 (2.1)	715 (0.8)
Cohabitation status:		
Living with the offspring’s father	769 582 (88.9)	72 514 (83.8)
Single	16 147 (1.9)	3433 (4.0)
Other	37 181 (4.3)	6346 (7.3)
Unknown	42 600 (4.9)	4258 (4.9)
Annual household income:		
1st quartile	205 433 (23.7)	27 828 (32.2)
2nd quartile	211 542 (24.4)	21 763 (25.1)
3rd quartile	213 782 (24.7)	19 513 (22.5)
4th quartile	216 696 (25.0)	16 732 (19.3)
Unknown	18 057 (2.1)	715 (0.8)
**Pregnancy characteristics**		
Parity:		
1	390 565 (45.1)	45 429 (52.5)
2-3	425 776 (49.2)	36 092 (41.7)
≥4	49 169 (5.7)	5030 (5.8)
Body mass index during early pregnancy, kg/m^2^:		
<18.5	19 430 (2.2)	1985 (2.3)
18.5-24	473 547 (54.7)	42 881 (49.5)
25-30	205 200 (23.7)	21 359 (24.7)
>30	100 400 (11.6)	13 324 (15.4)
Unknown	66 933 (7.7)	7002 (8.1)
Smoking during early pregnancy:		
No smoking	767 753 (88.7)	70 463 (81.4)
Yes, 1-9 cigarettes/day	40 236 (4.6)	8192 (9.5)
Yes, ≥10 cigarettes/day	11 181 (1.3)	3310 (3.8)
Unknown	46 340 (5.4)	4586 (5.3)
Hypertensive disorders:		
No	836 644 (96.7)	82 660 (95.5)
Gestational hypertension/pre-eclampsia:	11 604 (1.3)	1622 (1.9)
Pregestational	17 262 (2.0)	2269 (2.6)
Diabetic disorders:		
No	838 050 (96.8)	82 847 (95.7)
Gestational	11 872 (1.4)	1654 (1.9)
Pregestational	15 588 (1.8)	2050 (2.4)
Pre-existing psychiatric disorder:		
No	796 861 (92.1)	54 313 (62.8)
Depression	23 735 (2.7)	17 411 (20.1)
Other psychiatric disorder	44 914 (5.2)	14 827 (17.1)
Mode of delivery:		
Vaginal, not assisted	662 771 (76.6)	60 661 (70.1)
Vaginal, assisted	60 365 (7.0)	6480 (7.5)
Caesarean section	142 374 (16.4)	19 410 (22.4)
Gestational age, weeks:		
22-31	6405 (0.7)	1066 (1.2)
32-36	34 228 (4.0)	5062 (5.8)
37-40	599 436 (69.3)	62 078 (71.7)
≥41	225 441 (26.0)	18 345 (21.2)
Birth weight, g:		
<1500	5446 (0.6)	852 (1.0)
1500 to <2500	21 816 (2.5)	3152 (3.6)
2500 to <4200	746 174 (86.2)	74 027 (85.5)
≥4200	90 864 (10.5)	8347 (9.6)
Unknown	1210 (0.1)	173 (0.2)
Child loss:		
No	861 379 (99.5)	85 686 (99.0)
Stillbirth	2951 (0.3)	619 (0.7)
Infant death†	1180 (0.1)	246 (0.3)

IQR=interquartile range; BMI=body mass index.

* Country of birth: Nordic indicated women born in Nordic countries including Denmark, Finland, Iceland, Norway, and Sweden. Other/unknown referred to women born in other countries or with no information (<0.01%).

† Infant death referred to offspring died within first year after birth.

### All cause mortality

Over the follow up of up to 18 years (median 7.0 years), we identified 522 deaths (incidence rate 0.82 per 1000 person years) among women with perinatal depression and 1568 deaths (0.26) among women who did not have perinatal depression. Compared with the matched women in the control group, women with perinatal depression were associated with a three times higher risk of death (hazard ratio 3.26 (95% confidence interval 2.95 to 3.60); table 2, model 1). Following adjustment for pregnancy characteristics, the increased risk attenuated yet remained pronounced (2.11 (1.86 to 2.40) model 3). The association was similar when comparing mortality between full sisters (2.12 (1.16 to 3.88)). The complete case analysis yielded similar results (supplementary table S3). In the population matched cohort, increased mortality was associated with women who had no pre-existing psychiatric disorders (2.09 (1.79 to 2.45), model 3) and women who already had these disorders (2.16 (1.70 to 2.75), model 3). Similar patterns were indicated in the sibling comparison (1.98 (0.97 to 4.01) and for women without and with psychiatric history (2.23 (0.88 to 5.64). We noted the association of risk increase for both antepartum and postpartum depression (1.62 (1.34 to 1.94) and 2.71 (2.26 to 3.26)), with more of a pronounced association for postpartum depression (supplementary table S4 and S5).

**Table 2:** Hazard ratios (HRs) with 95% confidence intervals (CIs) for all cause mortality among patients with perinatal depression, compared with matched unaffected individuals or full siblings, overall and by pre-existing psychiatric disorder.

Overall association	Population matched cohort	Sibling cohort
No perinatal depression:		
No of deaths (rate*)	1568 (0.26)	694 (0.26)
Perinatal depression:		
No of deaths (rate*)	522 (0.82)	116 (0.62)
HR (95% CI), model 1†	3.26 (2.95 to 3.60)	2.69 (1.69 to 4.28)
HR (95% CI), model 2‡	2.88 (2.60 to 3.20)	2.46 (1.52 to 3.98)
HR (95% CI), model 3§	2.11 (1.86 to 2.40)	2.12 (1.16 to 3.88)
**With no pre-existing psychiatric disorder**		
No perinatal depression:		
No of deaths (rate*)	1386 (0.24)	621 (0.25)
Perinatal depression:		
No of deaths (rate*)	253 (0.60)	57 (0.46)
HR (95% CI), model 1†	2.50 (2.17 to 2.87)	2.22 (1.21 to 4.05)
HR (95% CI), model 2‡	2.34 (2.03 to 2.70)	2.07 (1.12 to 3.82)
HR (95% CI), model 3§	2.09 (1.79 to 2.45)	1.98 (0.97 to 4.01)
**With pre-existing psychiatric disorder**		
No perinatal depression:		
No of deaths (rate*)	182 (0.50)	73 (0.51)
Perinatal depression:		
No of deaths (rate*)	269 (1.24)	59 (0.93)
HR (95% CI), model 1†	2.41 (1.95 to 2.97)	2.16 (1.00 to 4.65)
HR (95% CI), model 2‡	2.22 (1.78 to 2.75)	2.10 (0.95 to 4.64)
HR (95% CI), model 3§	2.16 (1.70 to 2.75)	2.23 (0.88 to 5.64)

* Per 1000 person years, unadjusted.

† Maternal age and calendar year at delivery (ie, the matching factors) were inherently adjusted for in the population matched cohort; and were controlled for in the sibling cohort.

‡ Demographic characteristics including educational level, annual household income, country of birth (for population matched cohort), and cohabitation status were additional adjusted for.

§ Pregnancy characteristics including body mass index and smoking during early pregnancy, parity, pre-existing psychiatric disorder (for overall association), and diabetic and hypertensive disorders were additionally adjusted for.

### Temporal patterns

**Fig 1:**
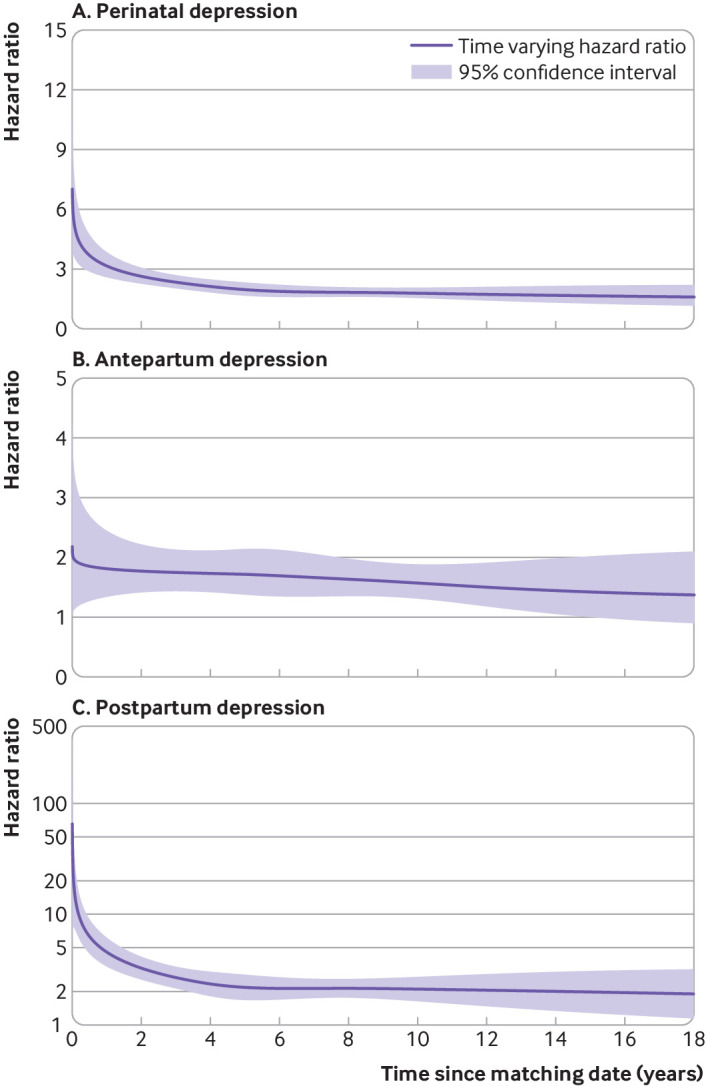
Hazard ratio (with 95% confidence interval) for all cause mortality among women with perinatal depression, compared with matched women with no perinatal depression. (A) Perinatal depression and (B) Antepartum depression, and (C) postpartum depression. *Time varying hazard ratios and 95% confidence intervals were derived from flexible parametric survival models allowing relative risk of perinatal depression to vary over time. A spline with five degrees of freedom was used for the baseline cumulative hazard, and three degrees of freedom was used for the time-varying effect. Models for perinatal and antepartum depression were adjusted for maternal age, calendar year at delivery, educational level, annual household income, country of birth, cohabitation status, parity, body mass index, smoking during early pregnancy, pre-existing psychiatric disorder, hypertensive and diabetic disorders. Model for postpartum depression was additionally controlled for delivery mode, gestational age, child loss, and birth weight

The cumulative rate of all cause mortality increased over time for women with perinatal depression and was associated with a considerably higher risk than in the matched women with no perinatal depression (supplementary figure S4). Women with perinatal depression appeared to have a much higher risk of mortality shortly after the perinatal depression diagnosis (fig 1; hazard ratio 5.01 (95% confidence interval 3.51 to 7.16) at 30 days after perinatal depression) compared with women who did not have perinatal depression. The increased risk reduced over time (3.16 (2.59 to 3.86) at one year) but remained higher 18 years later (1.60 (1.16 to 2.21)). A similar pattern was observed for postpartum depression, whereas the increased risk in mortality was less pronounced and relatively stable over time for antepartum depression. Similar results were obtained for period specific hazard ratios (supplementary table S6).

### Cause specific mortality

The increased risk associated with perinatal depression was greater for death caused by unnatural causes (incidence rate 0.46 for women with perinatal depression; hazard ratio 4.28 (95% confidence interval 3.44 to 5.32); table 3)than by natural causes (0.36; 1.38 (1.16 to 1.64)). Although suicide was rare (incidence rate 0.23), the increased risk of mortality due to suicide was most pronounced (hazard ratio 6.34 (95% confidence interval 4.62 to 8.71)), followed by mortality due to an accident (incidence rate 0.12; hazard ratio 3.10 (2.06 to 4.66)). The association was largely similar for women with and with no pre-existing psychiatric disorder. Associations appeared stronger for postpartum than for antepartum depression (supplementary table S7). The frequency of deaths due to suicide was particularly high during the immediate years after diagnosis, in particular after postpartum depression (supplementary fig s5). Regarding natural causes, a higher risk of cancer specific mortality was noted after perinatal depression (hazard ratio 1.32 (95% confidence interval 1.07 to 1.63)), particularly among women with no pre-existing psychiatric disorder (1.49 (1.18 to 1.89)) and after postpartum depression (1.80 (1.35 to 2.41)). Breast and cervical cancers were the leading reasons for cancer specific mortality (supplementary table S8). When women with a pre-existing cancer diagnosis were excluded, no association was noted (hazard ratio 0.99 (95% confidence interval 0.77 to 1.26)). We reported similar associations when women with a pre-existing cardiovascular disease were included and excluded (hazard ratios 1.72 *v* 1.83). Similar associations were also observed when additionally adjusting for pre-existing comorbidities (supplementary table S9).

**Table 3:** Hazard ratios (HRs) with 95% confidence intervals (CIs) for cause specific mortality among women with perinatal depression and by pre-existing psychiatric disorder, compared with matched individuals who were not affected, by common cause of death

Cause specific mortality	Overall			Women with no pre-existing psychiatric disorder			Women with pre-existing psychiatric disorder	
	No of deaths (rate*) in women with no perinatal depression/with perinatal depression	HR (95% CI) †		No of deaths (rate*) in women with no perinatal depression/with perinatal depression	HR (95% CI) †		No of deaths (rate*) women with no perinatal depression/with perinatal depression	HR (95% CI) †
Death due to unnatural cause:	305 (0.05)/290 (0.46)	4.28 (3.44 to 5.32)		233 (0.04)/113 (0.27)	4.83 (3.65 to 6.38)		72 (0.20)/177 (0.81)	3.43 (2.31 to 5.10)
Suicide	117 (0.02)/149 (0.23)	6.34 (4.62 to 8.71)		81 (0.01)/63 (0.15)	7.84 (5.24 to 11.73)		36 (0.10)/86 (0.40)	4.19 (2.31 to 7.60)
Accident	121 (0.02)/78 (0.12)	3.10 (2.06 to 4.66)		101 (0.02)/26 (0.06)	2.84 (1.67 to 4.83)		20 (0.05)/52 (0.24)	3.58 (1.71 to 7.53)
Others	67 (0.01)/63 (0.10)	5.83 (3.15 to 10.81)		51 (0.01)/24 (0.06)	7.37 (3.42 to 15.87)		16 (0.04)/39 (0.18)	3.96 (1.47 to 10.66)
Death due to natural cause:	1263 (0.20)/232 (0.36)	1.38 (1.16 to 1.64)		1153 (0.20)/140 (0.33)	1.46 (1.20 to 1.79)		110 (0.30)/92 (0.42)	1.17 (0.83 to 1.64)
Cancer	927 (0.15)/138 (0.22)	1.32 (1.07 to 1.63)		859 (0.15)/97 (0.23)	1.49 (1.18 to 1.89)		68 (0.19)/41 (0.19)	0.88 (0.56 to 1.39)
* * Excluding women with a history of cancer	822 (0.13)/97 (0.15)	0.99 (0.77 to 1.26)		764 (0.13)/64 (0.15)	1.11 (0.84 to 1.47)		58 (0.16)/33 (0.15)	0.76 (0.47 to 1.25)
Cardiovascular disease	134 (0.02)/30 (0.05)	1.72 (0.96 to 3.09)		125 (0.02)/15 (0.04)	1.35 (0.64 to 2.81)		9 (0.02)/15 (0.07)	3.07 (0.92 to 10.19)
Excluding women with a history of cardiovascular disease	113 (0.02)/ 26 (0.04)	1.83 (0.97 to 3.44)		106 (0.02)/15 (0.04)	1.69 (0.79 to 3.61)		7 (0.02)/11 (0.05)	2.11 (0.60 to 7.45)
Other	202 (0.03)/64 (0.10)	1.66 (1.12 to 2.48)		169 (0.03)/28 (0.07)	1.66 (1.01 to 2.76)		33 (0.09)/36 (0.17)	1.45 (0.74 to 2.88)

* Per 1000 person years, unadjusted.

† HRs for perinatal and antepartum depression were adjusted for educational level, annual household income, country of birth, cohabitation status, parity, smoking, and body mass index during early pregnancy, pre-existing psychiatric disorder (for overall association), diabetic, and hypertensive disorders.

### Additional analyses

Similar associations between perinatal depression and mortality were noted for clinical diagnosis versus use of antidepressants (supplementary table S10). Both age and calendar period modified the association (P for interaction <0.05; table 4). The risk elevation was greater in women younger than 30 years compared with older women, and in the period after perinatal depression screening (2010-17) compared with the period before (2001-09). For the screening periods, such a difference was not explained by the different length of follow-up (supplementary table S6). We did not find that associations differed substantially in relation to time of onset of perinatal depression since pregnancy or delivery (supplementary table S11).

**Table 4:** Hazard ratios (HRs) with 95% confidence intervals (CIs) for all cause mortality among patients with perinatal depression, compared with their matched unaffected individuals, stratified by age and calendar year at delivery, and pre-existing psychiatric disorder

All cause mortality	Perinatal depression			Antepartum depression			Postpartum depression	
	No of deaths (rate*) in women with no perinatal depression/with perinatal depression	HR (95% CI)†		No of deaths (rate*) in women with no perinatal depression/with perinatal depression	HR (95% CI)†		No of deaths (rate*) in women with no perinatal depression/with perinatal depression	HR (95% CI)†
Age at delivery, years:								
≤25	207 (0.18)/108 (0.91)	2.99 (2.26 to 3.94)		126 (0.20)/49 (0.76)	2.06 (1.39 to 3.05)		81 (0.15)/59 (1.10)	4.14 (2.75 to 6.22)
26-30	283 (0.15)/137 (0.73)	3.20 (2.50 to 4.10)		153 (0.15)/73 (0.69)	2.72 (1.92 to 3.86)		130 (0.16)/64 (0.79)	3.69 (2.58 to 5.29)
31-35	472 (0.24)/130 (0.65)	1.85 (1.47 to 2.34)		264 (0.24)/68 (0.60)	1.45 (1.04 to 2.03)		208 (0.24)/62 (0.72)	2.20 (1.57 to 3.08)
≥36	606 (0.47)/147 (1.11)	1.47 (1.18 to 1.84)		379 (0.49)/82 (1.02)	1.14 (0.85 to 1.53)		227 (0.44)/65 (1.25)	2.03 (1.46 to 2.82)
P for interaction‡	—	<0.001		—	0.001		—	0.008
Calendar year at delivery:								
2001-09	1189 (0.31)/354 (0.90)	1.90 (1.63 to 2.20)		717 (0.33)/194 (0.86)	1.53 (1.24 to 1.88)		472 (0.28)/160 (0.95)	2.34 (1.87 to 2.93)
2010-17	379 (0.16)/168 (0.69)	2.74 (2.21 to 3.39)		205 (0.15)/78 (0.56)	1.90 (1.38 to 2.62)		174 (0.17)/90 (0.87)	3.62 (2.69 to 4.86)
P for interaction‡		0.004			0.234			0.018

* Per 1000 person years, unadjusted.

† HRs for perinatal and antepartum depression were adjusted for educational level, annual household income, country of birth, cohabitation status, parity, smoking and body mass index during early pregnancy, pre-existing psychiatric disorder, diabetic, and hypertensive disorders. HRs for postpartum depression were additionally controlled for delivery mode, gestational age, birth weight, and child loss.

‡ An interaction term was included in the Cox regression models and P for interaction as examined by Wald test.

## Discussion

### Principal findings

In this nationwide, population based, matched cohort study of women in Sweden, we report that women with clinically diagnosed perinatal depression who attended specialised care or were treated by antidepressants, either antepartum or postpartum, are at an increased risk of mortality, independent of pre-existing psychiatric disorders. Observed associations cannot be explained by familial factors shared between full sisters. The increased mortality was most pronounced during the first year after perinatal depression diagnosis, although gradually attenuated, remained higher throughout the 18 years of follow up. Unnatural deaths and suicide, although rare, were particularly responsible for the highest relative risk.

### Comparison with other studies

In line with previous studies reporting a twofold to fivefold increase in mortality in women with postpartum psychiatric disorders,^6^ ^7^ ^8^ our results showed that women with postpartum depression had an almost threefold increased risk of mortality. This finding was not surprising because depression is one of the most common psychiatric disorders in the postpartum period.^9^ Increased relative risks remained from the sibling comparison, indicating that factors shared by sisters, such as potential childhood maltreatment^13^ ^15^ and parental psychiatric morbidity,^12^ ^14^ are unlikely to explain the observed association. The association was largely similar among women with and with no pre-existing depression or other psychiatric disorder, suggesting that perinatal depression associated mortality risk is independent of psychiatric history. This highlights the need to include women with perinatal depression and who have a history of psychiatric disorders in future perinatal depression studies, due to the additive risk of mortality from other psychiatric disorder associated risk. Because perinatal depression diagnosed in primary care and not treated with antidepressants are not covered in this study, the incidence of perinatal depression and its association with mortality is likely to be underestimated. Importantly, our study fills a knowledge gap in finding an increased mortality among women with antepartum depression, albeit less pronounced than postpartum depression. This may partly be explained by differences in suicidal behaviour, of which the risks are greater in the postpartum period compared with the antepartum period,^5^ ^11^ possibly due to decreased mental health treatment options for pregnant women,^34^ which may not result in a live birth to be studied in research. However, the underlying mechanisms remain unclear.

A previous study reported that women with postpartum psychiatric disorders had a 19-fold increased risk in all cause mortality during the first year following childbirth.^7^ To provide evidence on actionable time windows, we visualised the time varying association by time points and periods, and both showed that the elevated mortality was greatest shortly after the diagnosis of perinatal depression, particularly for postpartum depression. For instance, one week after diagnosis, women with postpartum depression had an almost sixfold risk of death. The elevated mortality attenuated but remained higher (hazard ratio >3) throughout the first year after diagnosis. This finding is consistent with previous reports of two to five times increased risk of mortality associated with psychiatric disorders one year post partum,^6^ ^7^ ^8^ with the highest risk noted closely after the perinatal depression diagnosis.^6^ Although our results should not be interpreted as from the onset of perinatal depression, the findings highlight that, in clinical practice, the immediate period after a postpartum depression diagnosis could be crucial for prevention. Building on previous findings,^7^ we further show that risk of mortality was about twice as high 18 years after postpartum depression in women with this disorder. For women with antepartum depression however, we reported that such an association existed, although the risk was more modest. A twofold elevated risk in the first year from diagnosis attenuated slowly and gradually throughout follow-up. Women with perinatal depression are associated with increased negative health behaviours (eg, unhealthy diet and increased substance use) and comorbidities (eg, long term mood disorders), and therefore, had higher risk of death decades after perinatal depression diagnosis. Although the increased risk of mortality 10 years later may not be a direct result of perinatal depression, our findings may help healthcare providers to predict and identify mothers at high risk in the long term.

Risks of maternal suicide during the perinatal period are well documented.^2^ ^34^ Nearly half of the cases were of women who had a depression diagnosis,^35^ although the measurement on suicide remains less accurate^34^ and often is studied with other outcomes (eg, accident) together.^6^ Consistent with previous studies,^6^ ^7^ we found that suicide mortality, although rare, yielded the highest risk elevations. Emerging data have also shown that women with PND are at increased risk of suicide attempt.^36^ Previous studies have often investigated deaths due to unnatural causes as a combined category, including accidents, suicides, and homicides, most likely due to small sample size.^6^ ^7^ ^8^ Our data are the first, to our knowledge, to show that women with perinatal depression were at an increased risk of death due to accidents alone. Given the preventable nature, albeit rareness, of suicide and accidents, the strong associations particularly for postpartum depression (for suicide, incidence rate 0.23 and hazard ratio 12.17; for accident specific mortality, 0.12 and 6.07) call for targeted urgent actions to address the unmet needs of women with perinatal depression who are at high risk in maternal healthcare. Additionally, we report an increased risk of mortality due to natural causes after rigorous adjustment for somatic comorbidities. Cancer mortality has been linked to depression occurring at any time in life^37^ and to postpartum psychiatric disorders.^8^ The association between postpartum depression and cancer specific mortality might be explained due to the increased risk of depression following a recent cancer diagnosis.^38^ In support of this association, we found no association of perinatal depression with cancer specific mortality when women with pre-existing cancer were excluded. However, we also observed a similar result by excluding women with pre-existing cardiovascular disease or by stratifying on pre-existing psychiatric disorder. This lends support to the fact that our findings were not completely explained by a group that is high risk and captured by a perinatal depression diagnosis. In addition, perinatal depression has been linked to other somatic diseases, for example, autoimmune disease.^39^ Future studies on deaths due to other less common natural causes are warranted.

### Strengths and limitations of this study

Our study takes advantage of a large nationwide matched cohort using prospectively and independently collected data that enable a complete follow-up of study participants. The large sample size allowed us to explore cause specific mortality, even for uncommon causes (eg, cancer mortality). Additionally, we were able to control for various confounding factors, including the use of sibling comparison to address shared familial factors.

However, some limitations should be acknowledged. Firstly, perinatal depression was identified through clinical diagnosis recorded in the inpatient and outpatient specialised care, as well as through dispensed antidepressants. Hence, we missed women with mild to moderate perinatal depression who did not use antidepressants and only sought help through primary care, other support systems, or not at all. Although no direct estimate for perinatal depression is available, fewer than 30% of patients with depression or anxiety attended by primary care were not prescribed medications.^40^ Classifying mild perinatal depression into the reference group would have led to attenuated associations. Until 2010, no official scale was used to screen for perinatal depression; we found the association between perinatal depression and mortality to be more pronounced from 2010, when the screening using the Edinburgh postnatal depression scale was introduced nationwide.^41^ Before using this scale, diagnosis of perinatal depression mostly relied on the experiences of frontline professionals and women with this disorder, therefore, cases of perinatal depression were more likely to have been missed. Secondly, antidepressants may be prescribed for other indications than depression. Reassuringly, similar results were obtained when relying on clinical diagnosis of perinatal depression alone. Thirdly, we missed maternal deaths occurred during pregnancy if not resulting in a birth. However, such cases are very few, for example, suicide during pregnancy only accounts for 2.9% of all perinatal suicide in Sweden.^11^ Such misclassification should have attenuated the association for antepartum depression and not affect the results for postpartum depression. Fourthly, we may have misclassified some suicide events as accidents because only deaths with clear evidence of suicidal intention were coded as suicide, which might explain the higher risk of mortality due to accidents. This misclassification, if any, might have attenuated the point estimate for suicide, although the validity for unnatural cause of death is high in the register.^19^ Additionally, the hazard ratio derived from Cox regression is an average estimate over time since perinatal depression. The time varying hazard ratios from flexible parametric modelling are more accurate and not subject to the proportional hazard assumption. Although we have comprehensively controlled for potential confounders through adjustment and sibling comparison, we cannot exclude the possibility of residual confounding due to unknown and unmeasured confounders that are not necessarily shared between full siblings (eg, physical activity or domestic violence). Lastly, our findings shall be generalised with cautions to women not covered by this study (eg, mild perinatal depression or multiple gestations), or to other populations with different cultural backgrounds or healthcare systems.

### Conclusions

Women with clinically diagnosed perinatal depression are at an increased risk of mortality independent of psychiatric history and familial factors. The association is stronger for unnatural death, particularly due to suicide, and during the first year after diagnosis. Women’s background characteristics explained a large part, but not all, of the observed association. Women affected by perinatal depression, their families, and health professionals, particularly those working in primary, maternal, and mental care, need to be aware of the serious health hazards regardless of psychiatric history. Early detection and treatment are needed for groups at high risk of perinatal depression to prevent the fatal outcomes.

**What is already known on this topic**

Perinatal depression is one of the most common complications of pregnancy, affecting up to 20% women around delivery
Women with postpartum psychiatric disorders, including psychotic, affective, and anxiety disorders have an increased risk of death compared with mothers who are unaffected or the general female population
Factors shared within families might contribute to the increased mortality, however, the evidence is limited

**What this study adds**

Women with perinatal depression are at an increased risk of mortality, independent of pre-existing psychiatric disorders, and particularly due to suicide and during the first year after diagnosis
Observed associations cannot be explained by familial factors shared between full sisters

## Data Availability

The register data are not publicly available due to privacy protection, including General Data Protection Regulation (GDRP). Access to Swedish register resources is only granted after ethical review by appropriate authorities. Further information on data access to Swedish register data can be found at the Swedish National Board of Health and Welfare (https://bestalladata.socialstyrelsen.se/, email: registerservice@socialstyrelsen.se) and/or Statistics Sweden (https://www.scb.se/vara-tjanster/bestall-data-och-statistik/, email: scb@scb.se). Codes on data analyses can be shared on request to the corresponding author at (qingshen@tongji.edu.cn).

## Web extra

material supplied by authors

Web appendix: Extra material

## Notes

Contributors: DL and QS are senior authors who contributed equally to this study. DL can be contacted on Twitter via @LuDonghao. DL and QS had full access to all the data in this study and take full responsibility for the integrity of the data and the accuracy of the data analysis. DL conceived and designed the study. DL, QS, and NH undertook the statistical analysis. DL, QS, NH, EB, ASO, and UAV drafted the manuscript. DL and QS are the guarantors. All authors provided critical input to the analyses, interpreted the data, and revised the manuscript critically. The corresponding authors attest that all listed authors meet authorship criteria and that no others meeting the criteria have been omitted.

The lead authors (DL, QS, and NH) affirm that the manuscript is an honest, accurate, and transparent account of the study being reported; that no important aspect of the study have been omitted; and that any discrepancies from the study as planned have been explained.

Dissemination to participants and related patient and public communities: Dissemination to the Swedish population (which constitutes the study population) will be through media outreach (eg, press release and communication) on publication of this study.

Provenance and peer review: Not commissioned; externally peer reviewed.

## References

[1] O’Hara MW Wisner KL . Perinatal mental illness: definition, description and aetiology. Best Pract Res Clin Obstet Gynaecol 2014;28:3-12. 10.1016/j.bpobgyn.2013.09.002. 24140480 PMC7077785

[2] Gavin NI Gaynes BN Lohr KN Meltzer-Brody S Gartlehner G Swinson T . Perinatal depression: a systematic review of prevalence and incidence. Obstet Gynecol 2005;106:1071-83. 10.1097/01.AOG.0000183597.31630.db. 16260528

[3] American Psychiatric Association . Diagnostic and Statistical Manual of Mental Disorders (Fifth ed). Arlington, Virginia: American Psychiatric Publishing.

[4] Lee Y-L Tien Y Bai Y-S . Association of postpartum depression with maternal suicide: a nationwide population-based study. Int J Environ Res Public Health 2022;19:5118. 10.3390/ijerph19095118. 35564525 PMC9099720

[5] Lindahl V Pearson JL Colpe L . Prevalence of suicidality during pregnancy and the postpartum. Arch Womens Ment Health 2005;8:77-87. 10.1007/s00737-005-0080-1. 15883651

[6] Johannsen BMW Larsen JT Laursen TM Bergink V Meltzer-Brody S Munk-Olsen T . All-cause mortality in women with severe postpartum psychiatric disorders. Am J Psychiatry 2016;173:635-42. 10.1176/appi.ajp.2015.14121510. 26940804 PMC5325688

[7] Appleby L Mortensen PB Faragher EB . Suicide and other causes of mortality after post-partum psychiatric admission. Br J Psychiatry 1998;173:209-11. 10.1192/bjp.173.3.209. 9926095

[8] Johannsen BMW Laursen TM Bech BH Munk-Olsen T . General medical conditions and mortality in women with postpartum psychiatric disorders. Acta Psychiatr Scand 2020;142:467-75. 10.1111/acps.13232. 32918276

[9] Toohey J . Depression during pregnancy and postpartum. Clin Obstet Gynecol 2012;55:788-97. 10.1097/GRF.0b013e318253b2b4. 22828111

[10] Putnam KT Wilcox M Robertson-Blackmore E Postpartum Depression: Action Towards Causes and Treatment (PACT) Consortium . Clinical phenotypes of perinatal depression and time of symptom onset: analysis of data from an international consortium. Lancet Psychiatry 2017;4:477-85. 10.1016/S2215-0366(17)30136-0. 28476427 PMC5836292

[11] Esscher A Essén B Innala E . Suicides during pregnancy and 1 year postpartum in Sweden, 1980-2007. Br J Psychiatry 2016;208:462-9. 10.1192/bjp.bp.114.161711. 26494874

[12] Bauer AE Maegbaek ML Liu X . Familiarity of psychiatric disorders and risk of postpartum psychiatric episodes: a population-based cohort study. Am J Psychiatry 2018;175:783-91. 10.1176/appi.ajp.2018.17111184. 29730937 PMC6070397

[13] Choi KW Sikkema KJ . Childhood Maltreatment and Perinatal Mood and Anxiety Disorders: A Systematic Review. Trauma Violence Abuse 2016;17:427-53. 10.1177/1524838015584369. 25985988

[14] Qin P Agerbo E Mortensen PB . Suicide risk in relation to family history of completed suicide and psychiatric disorders: a nested case-control study based on longitudinal registers. Lancet 2002;360:1126-30. 10.1016/S0140-6736(02)11197-4. 12387960

[15] Segal L Armfield JM Gnanamanickam ES . Child maltreatment and mortality in young adults. Pediatrics 2021;147:e2020023416. 10.1542/peds.2020-023416. 33318224

[16] Cnattingius S Ericson A Gunnarskog J Källén B . A quality study of a medical birth registry. Scand J Soc Med 1990;18:143-8. 10.1177/140349489001800209. 2367825

[17] Ludvigsson JF Andersson E Ekbom A . External review and validation of the Swedish national inpatient register. BMC Public Health 2011;11:450. 10.1186/1471-2458-11-450. 21658213 PMC3142234

[18] Wettermark B Hammar N Fored CM . The new Swedish Prescribed Drug Register—opportunities for pharmacoepidemiological research and experience from the first six months. Pharmacoepidemiol Drug Saf 2007;16:726-35. 10.1002/pds.1294. 16897791

[19] Brooke HL Talbäck M Hörnblad J . The Swedish cause of death register. Eur J Epidemiol 2017;32:765-73. 10.1007/s10654-017-0316-1. 28983736 PMC5662659

[20] Richardson DB . An incidence density sampling program for nested case-control analyses. Occup Environ Med 2004;61:e59. 10.1136/oem.2004.014472. 15550597 PMC1740694

[21] Ekbom A . The Swedish Multi-generation Register. In: Dillner J , ed. Methods in Biobanking. Humana Press, 2011: 215-20, 10.1007/978-1-59745-423-0_10. 20949391

[22] Viktorin A Meltzer-Brody S Kuja-Halkola R . Heritability of perinatal depression and genetic overlap with nonperinatal depression. Am J Psychiatry 2016;173:158-65. 10.1176/appi.ajp.2015.15010085. 26337037

[23] Høgberg U Larsson N . Early dating by ultrasound and perinatal outcome. A cohort study. Acta Obstet Gynecol Scand 1997;76:907-12. 10.3109/00016349709034900. 9435727

[24] Fazel S Wolf A Chang Z Larsson H Goodwin GM Lichtenstein P . Depression and violence: a Swedish population study. Lancet Psychiatry 2015;2:224-32. 10.1016/S2215-0366(14)00128-X. 26236648 PMC4520382

[25] Noble RE . Depression in women. Metabolism 2005;54(Suppl 1):49-52. 10.1016/j.metabol.2005.01.014. 15877314

[26] Wang E Glazer KB Howell EA Janevic TM . Social determinants of pregnancy-related mortality and morbidity in the united states: a systematic review. Obstet Gynecol 2020;135:896-915. 10.1097/AOG.0000000000003762. 32168209 PMC7104722

[27] Grandi SM Filion KB Yoon S . Cardiovascular disease-related morbidity and mortality in women with a history of pregnancy complications. Circulation 2019;139:1069-79. 10.1161/CIRCULATIONAHA.118.036748. 30779636

[28] Walker ER McGee RE Druss BG . Mortality in mental disorders and global disease burden implications: a systematic review and meta-analysis. JAMA Psychiatry 2015;72:334-41. 10.1001/jamapsychiatry.2014.2502. 25671328 PMC4461039

[29] Lancaster CA Gold KJ Flynn HA Yoo H Marcus SM Davis MM . Risk factors for depressive symptoms during pregnancy: a systematic review. Am J Obstet Gynecol 2010;202:5-14. 10.1016/j.ajog.2009.09.007. 20096252 PMC2919747

[30] Tuohy A McVey C . Experience of pregnancy and delivery as predictors of postpartum depression. Psychol Health Med 2008;13:43-7. 10.1080/13548500701294531. 18066918

[31] Clark SL Belfort MA Dildy GA Herbst MA Meyers JA Hankins GD . Maternal death in the 21st century: causes, prevention, and relationship to Caesarean delivery. Am J Obstet Gynecol 2008;199:P36.e1-6. 10.1016/j.ajog.2008.03.007. 18455140

[32] Lambert PC Royston P . Further development of flexible parametric models for survival analysis. Stata J Promot Commun Stat Stata 2009;9:265-90. 10.1177/1536867X0900900206.

[33] Ludvigsson JF Appelros P Askling J . Adaptation of the Charlson Comorbidity Index for register-based research in Sweden. Clin Epidemiol 2021;13:21-41. 10.2147/CLEP.S282475. 33469380 PMC7812935

[34] Chin K Wendt A Bennett IM Bhat A . Suicide and maternal mortality. Curr Psychiatry Rep 2022;24:239-75. 10.1007/s11920-022-01334-3. 35366195 PMC8976222

[35] Khalifeh H Hunt IM Appleby L Howard LM . Suicide in perinatal and non-perinatal women in contact with psychiatric services: 15 year findings from a UK national inquiry. Lancet Psychiatry 2016;3:233-42. 10.1016/S2215-0366(16)00003-1. 26781366

[36] Yu H Shen Q Bränn E . Perinatal depression and risk of suicidal behavior. JAMA Netw Open [forthcoming]. 10.1001/jamanetworkopen.2023.50897 PMC10777256 38194232

[37] Pinquart M Duberstein PR . Depression and cancer mortality: a meta-analysis. Psychol Med 2010; 40:1797-810. 10.1017/S0033291709992285. 20085667 PMC2935927

[38] Lu D Andersson TML Fall K . Clinical diagnosis of mental disorders immediately before and after cancer diagnosis: a nationwide matched cohort study in Sweden. JAMA Oncol 2016;2:1188-96. 10.1001/jamaoncol.2016.0483. 27124325

[39] Bränn E Chen Y Song H . Bidirectional association between autoimmune disease and perinatal depression: a nationwide study with sibling comparison. Mol Psyc [forthcoming]. 10.1038/s41380-023-02351-1. PMC11153129 38191927

[40] Socialstyrelsens . Nationella riktlinjer för vård vid depression och ångestsyndrom. https://www.socialstyrelsen.se/globalassets/sharepoint-dokument/artikelkatalog/nationella-riktlinjer/2021-4-7339.pdf

[41] Reuter A . Barnhälsovårdens nationella program. Rikshandboken Barnhälsovård Hämtad. 2018. https://www.rikshandboken-bhv.se/metoder-riktlinjer/barnhalsovardens-nationella-program/

