# Supplementary material for: Perinatal depression and risk of mortality: nationwide, register based study in Sweden
Source: BMJ. 2024 Jan 10;384:e075462. doi: 10.1136/bmj-2023-075462 (PMC10777893; doi:10.1136/bmj-2023-075462)
Supplement: Supplementary file 1 — Web appendix: Extra material [file hagn075462.ww.pdf]

Supplementary material 1

Figure S1. Study design

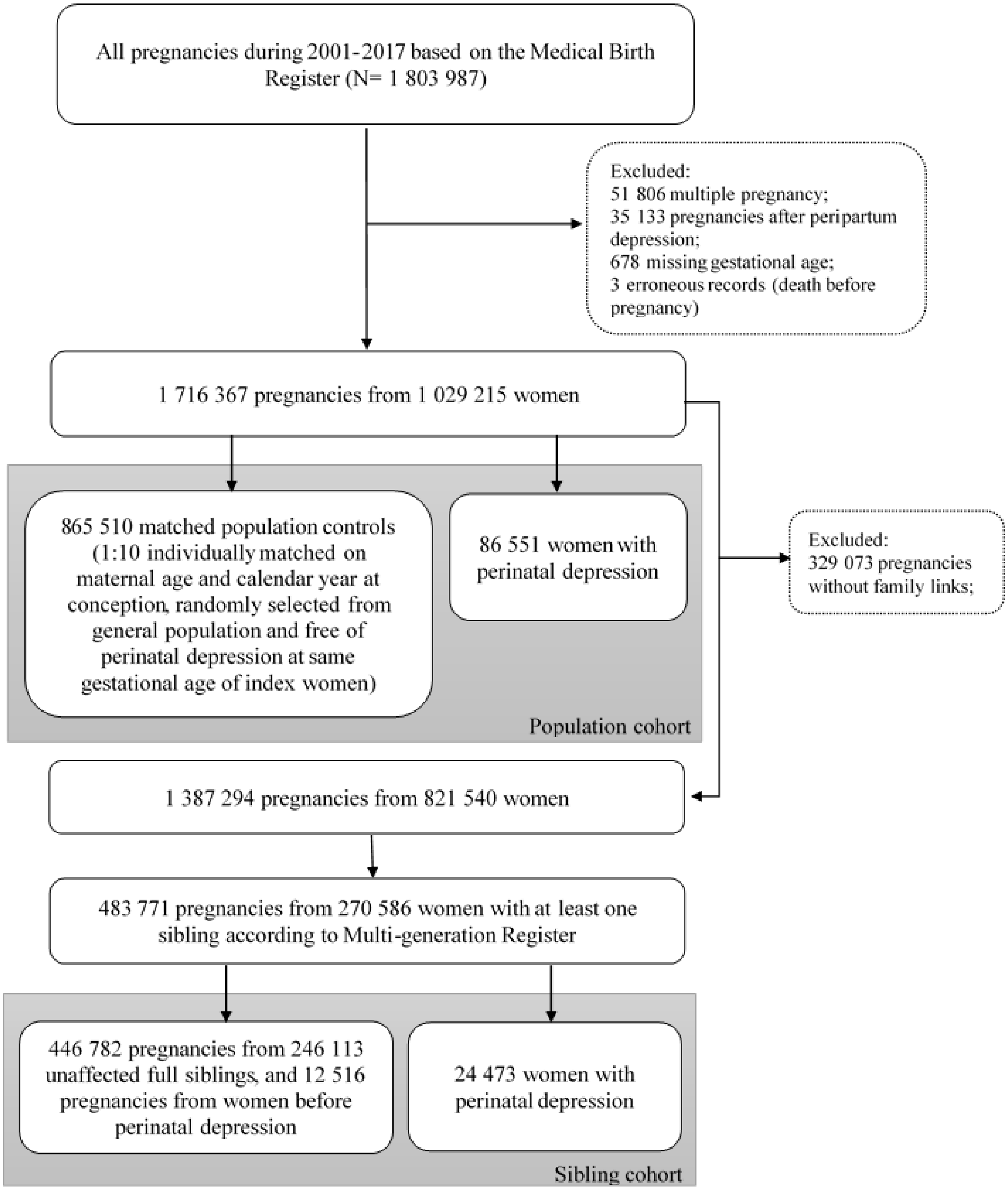

Figure S2. Follow-up for women with perinatal depression, their matched reference women and their full siblings.

A. Population cohort

For women with antepartum depression and their matched reference group

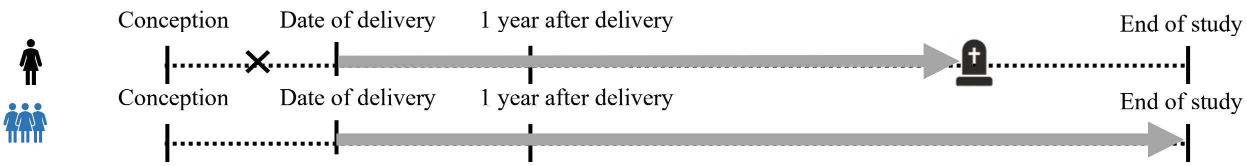

For women with postpartum depression and matched reference group

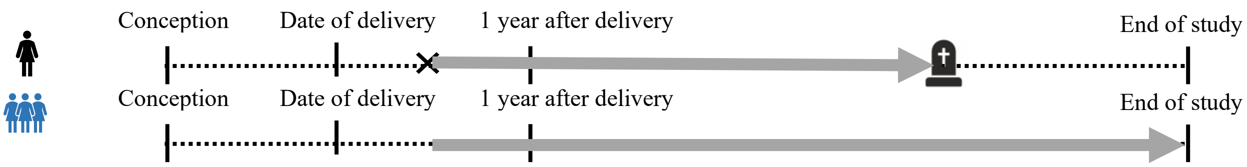

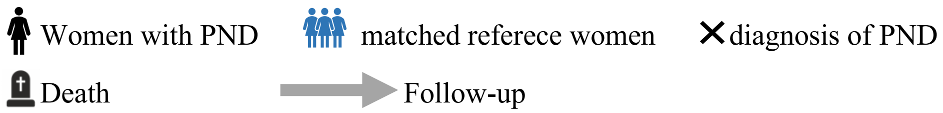

Women with PND and their matched reference women without PND were all selected from the study base, and were individually matched at the same gestational age (for antepartum depression) or postpartum days (for postpartum depression). Date of PND diagnosis or matching was used as index date. Women were followed from the index date (for women with postpartum depression and matched women) or from the delivery date (for women with antepartum depression and matched women), until emigration (from the Migration Register), death, a diagnosis of PND (in reference group), or December 31, 2018, whichever occurred first. Women were followed from the index date or from the delivery date, whichever came later, because women became at risk from the delivery date (for women with antepartum depression) and from the index date (for women with postpartum depression).

B. Sibling cohort

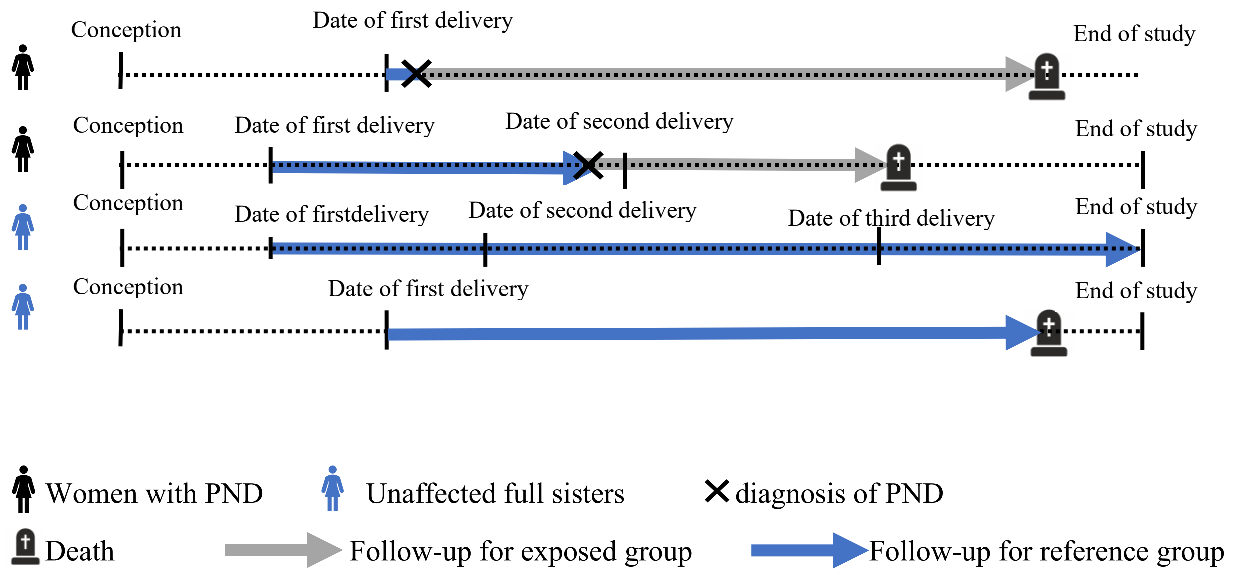

Sisters ever with live singleton births recorded in the Medical Birth Register and with identifiable parents tracked through the Multi-generation Register were included. We followed all full sisters from the date of their first delivery until emigration, death or December 31 2018, whichever came first. Specifically, women with a PND diagnosis (N= 24 473 women) contributed their person-time to the reference group from the date of first delivery, and to the exposed group when receiving a PND diagnosis. Unaffected full sisters (N= 246 113 women) contributed their person-time to the reference group from the date of first delivery. Multiple full sisters, if available, were all included in this analysis (31.7%).

Figure S3. Directed Acyclic Graph (DAG) for perinatal depression and risk of mortality.

A. Antepartum depression

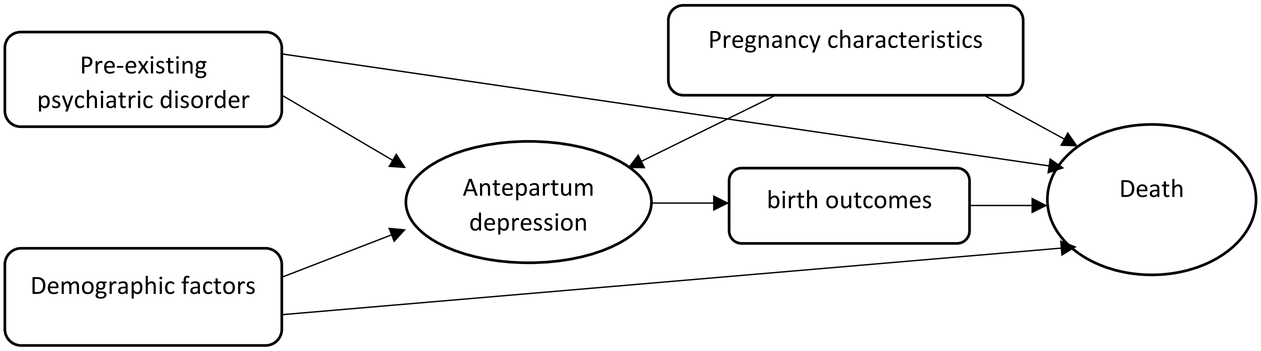

B. Postpartum depression

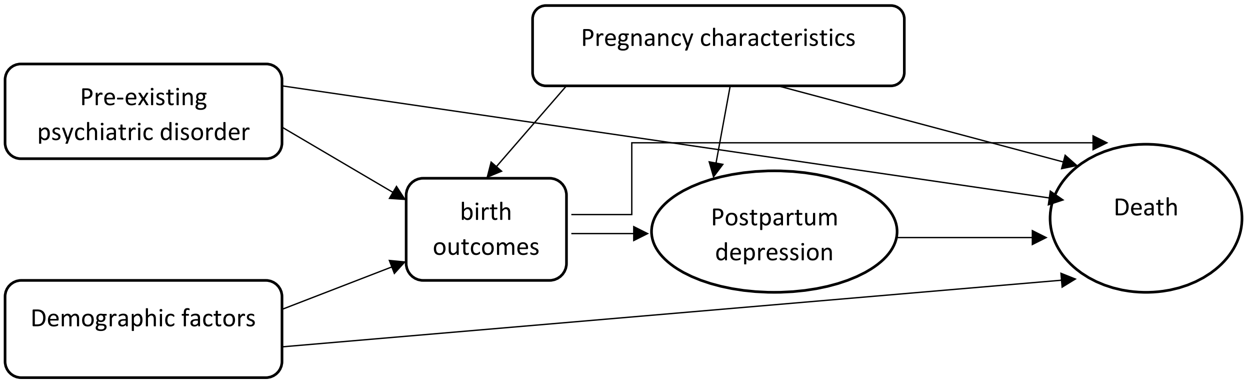

Demographic characteristics: maternal age, year at delivery, educational level, annual household income, country of birth (for population-matched cohort) and cohabitation status;

Pregnancy characteristics: BMI and smoking during early pregnancy, parity, and diabetic and hypertensive disorders;

Birth outcomes: develiry mode, gastational age, birth weight and child loss;

Figure S4. Cumulative all-cause and cause-specific mortality rates (per 1 000 person-years) among patients with and without perinatal depression over time.

A. All-cause mortality rate

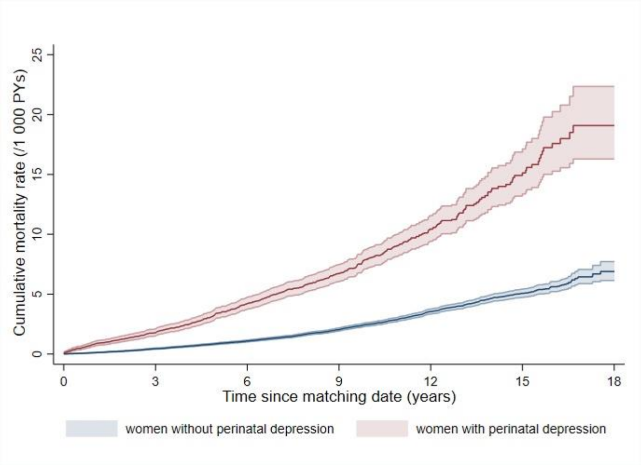

B. Cause-specific mortality rate – unnatural causes

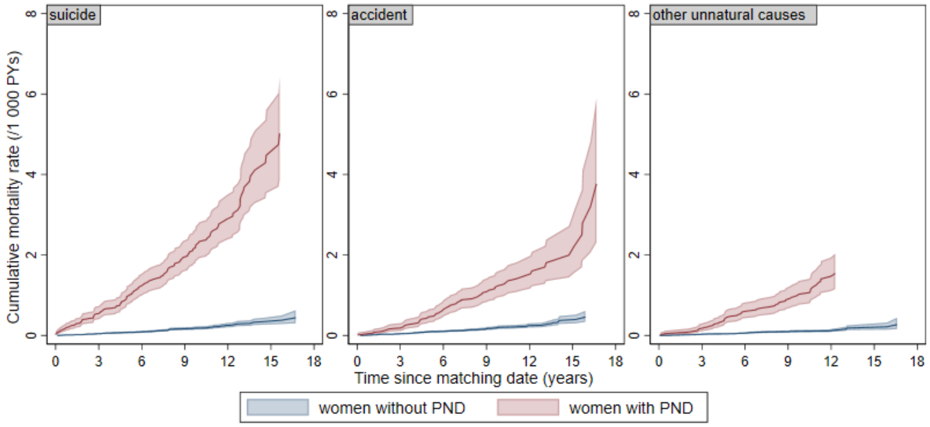

C. Cause-specific mortality rate – natural causes

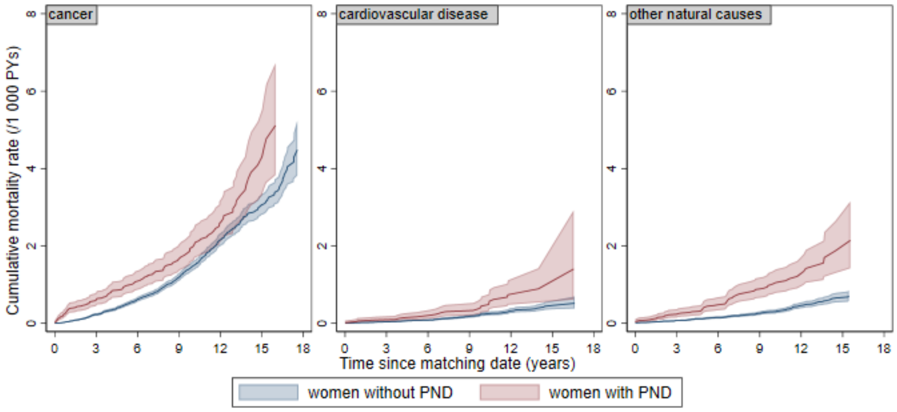

Figure S5 Frequency of death due to suicide and accident among women with perinatal depression over time

A. Suicide

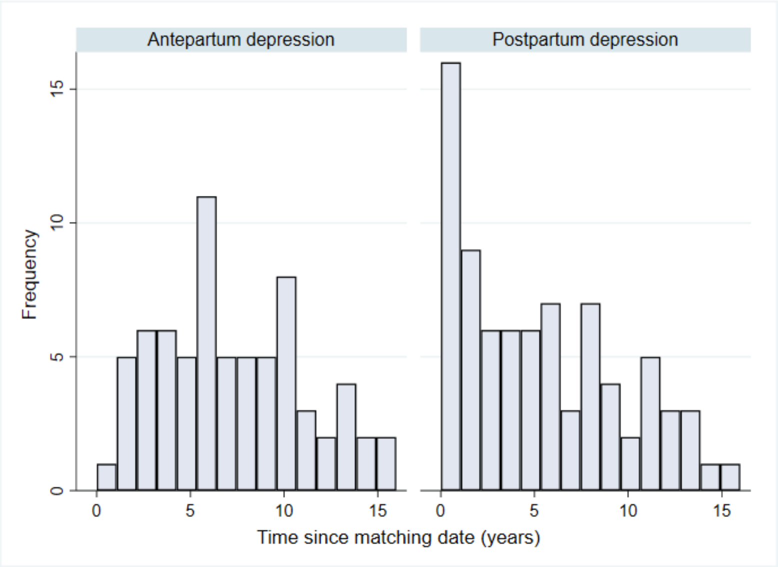

B. Accident

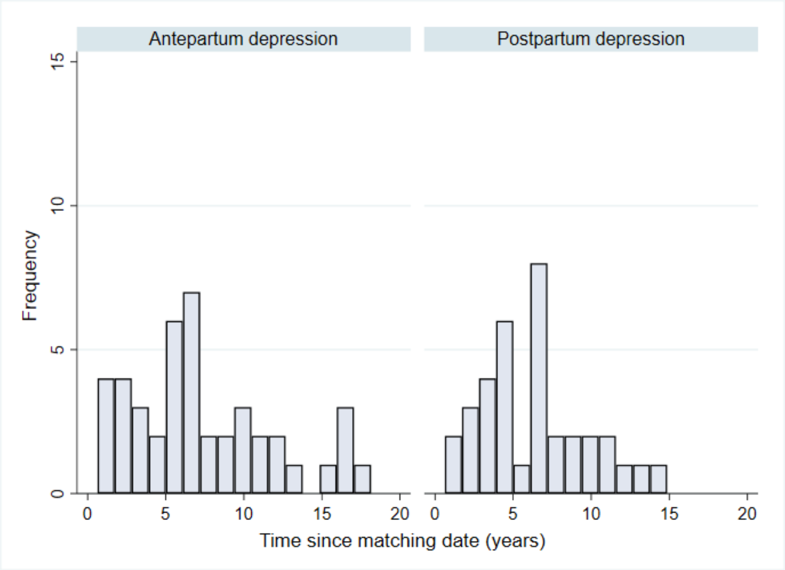

Table S1 Identification of perinatal depression, common causes of deaths and definitions for identifying related covariates.

	ICD 10
**Perinatal depression**	
Diagnoses from the Patient Register or MBR	F32.0, F32.1, F32.2, F32.3, F32.8, F32.9, F33.0, F33.1, F33.2, F33.3, F33.4, F33.8, F33.9 and F53.0
	**ATC**
Antidepressants from the Drug Register or MBR prescription	N06A
	**ICD-10**
**Cause of deaths**	
Overall	All chapters
Unnatural cause	V01-Y98
Suicide	X60-X84, Y870
Accident	V01-X59, Y85-Y86
Natural cause	A00-R99
Cancer	C00-C99
Cardiovascular disease	I00-I99
**Educational level and annual household income**	retrieved from the closest year near delivery from the LISA
**Child loss**	stillbirth from the MBR or death within first year after birth, identified through the Causes of Death Register

	ICD 8	ICD 9	ICD 10
**Pre-existing psychiatric disorder**			F10-F69
- Depression	-	296,298,300,311	F32-F33
- Other psychiatric disorder	-	291,292,295,297,299,301-311	F10-F31,F34-F69
**Hypertensive disorder**			
- Gestational	-	642D-642G, 642X	O12, O16
- Hypertension/preeclampsia	401-405	642C-642H	O10-O11, I10-I15
**Diabetic disorder**			
- Gestational	-	648W	O244
- Pregestational	250	648A	E10-E14, O241-O243

ATC, The Anatomical Therapeutic Chemical; ICD, International Classification of Diseases; MBR, Medical Birth Register; LISA, Longitudinal Integration Database for Health Insurance and Labor Market.

1. Perinatal depression

PND was defined as any diagnosis of depression or dispensation of antidepressants during pregnancy and up to one year after delivery, recorded in the MBR, NPR, or Prescribed Drug Register. The gestational age was, whenever possible, estimated from the routine ultrasound examination offered to all pregnant women at around gestational week 18-19 since 1990 and

performed in 95% of all pregnancies. Start of pregnancy was defined as the date obtained from subtracting the estimated gestational age from the delivery date. We considered both primary and secondary diagnoses to identify PND cases, using the 10th Swedish revision of the International Classification of Diseases (ICD-10) codes. Although a validity study has not been carried out specifically for PND, the diagnosis of depression has been validated in the NPR. To capture diagnoses made in primary care, we also identified use of antidepressant medication from maternal reports in the MBR and dispensations in the Prescribed Drug Register using the Anatomical Therapeutic Chemical (ATC) code as a proxy for PND.

The incident date of PND was defined as the date of the first recorded diagnosis or the prescribing date of a filled antidepressant, whichever came first. If a diagnosis of depression or self-report of antidepressant use was present in the MBR, the date corresponding to the individual median gestational length was assigned as the index date. The index date was then used to classify PND into ante- and postpartum depression.

**ICD-10-SE for Perinatal depression**	**Clinical Diagnosis**
F32.0	Mild depressive episode
F32.1	Moderate depressive episode
F32.2	Severe depressive episode without psychotic symptoms
F32.3	Severe depressive episode with psychotic symptoms
F32.8	Other specified depressive episodes
F32.9	Depressive episode, unspecified
F33.0	Recurrent depression, mild episode
F33.1	Recurrent depression, moderate episode
F33.2	Recurrent depression, severe episode without psychotic symptoms
F33.3	Recurrent depression, severe episode with psychotic symptoms
F33.4	Recurrent depression without current symptoms
F33.8	Other specified recurrent depressions
F33.9	Recurrent depression, unspecified
F53.0	Mild mental and behavioural disorders related to puerperium, not elsewhere classified: Postpartum depression UNS, Puerperal depression UNS

2. Covariates

We obtained information on demographics (i.e., maternal age at delivery, cohabitation status and country of birth) from the MBR and socioeconomic status (i.e., educational level and annual household income from the latest recorded year prior to the index date) from the Longitudinal Integration Database for Health Insurance and Labor Market (LISA), as known risk factors for both depression and premature death. Pregnancy-specific characteristics such as parity, body mass index (BMI), tobacco smoking, pregnancy complications (hypertensive and diabetic disorders), and pre-existing psychiatric disorders (any time before pregnancy) are putative risk factors for both PND and mortality, and were extracted from the MBR (NPR for pre-existing psychiatric disorders). Adverse birth outcomes could influence the risk of postpartum depression and maternal death, hence information on mode of delivery, gestational age, birth weight and stillbirth was obtained from the MBR. Child loss within first year after birth was identified through the Causes of Death Register.

Table S2. Baseline characteristics of women with and without perinatal depression in the sibling cohort, N (%).

		No perinatal depression N=459,298	Perinatal depression N=24,473
Demographic characteristics			
Age at delivery, years	≤20	9,089 (2.0%)	498 (2.0%)
	21-25	66,005 (14.4%)	3,782 (15.5%)
	26-30	158,900 (34.6%)	7,832 (32.0%)
	31-35	158,004 (34.4%)	8,054 (32.9%)
	36-40	59,359 (12.9%)	3,670 (15.0%)
	41-45	7,711 (1.7%)	628 (2.6%)
	≥46	230 (0.1%)	9 (0.0%)
Maternal year of birth, years,	≤1964	3,549 (0.8%)	120 (0.5%)
	1965-1969	26,505 (5.8%)	950 (3.9%)
	1970-1974	91,194 (19.9%)	3,517 (14.4%)
	1975-1979	132,016 (28.7%)	6,342 (25.9%)
	1980-1984	118,517 (25.8%)	7,136 (29.2%)
	1985-1989	68,111 (14.8%)	4,791 (19.6%)
	1990-1994	18,275 (4.0%)	1,506 (6.2%)
	≥1995	1,131 (0.2%)	111 (0.5%)
Country of birth[a]	Nordic	427,665 (93.1%)	23,105 (94.4%)
	Other/unknow	31,633 (6.9%)	1,368 (5.6%)
Educational level, years	≤9	37,557 (8.2%)	3,463 (14.2%)
	10-12	186,128 (40.5%)	10,381 (42.4%)
	>12	234,963 (51.2%)	10,581 (43.2%)
	Unknown	650 (0.1%)	48 (0.2%)
Cohabitation status	Living with the offspring's father	417,063 (90.8%)	20,814 (85.0%)
	Single	5,602 (1.2%)	867 (3.5%)
	Other	14,232 (3.1%)	1,604 (6.6%)
	Unknown	22,401 (4.9%)	1,188 (4.9%)
Annual household income	1st quartile	112,669 (24.5%)	8,071 (33.0%)
	2nd quartile	115,107 (25.1%)	5,669 (23.2%)
	3rd quartile	115,180 (25.1%)	5,574 (22.8%)
	4th quartile	115,692 (25.2%)	5,111 (20.9%)
	Unknown	650 (0.1%)	48 (0.2%)
Pregnancy characteristics			
Parity	1	206,401 (44.9%)	12,860 (52.5%)
	2-3	233,931 (50.9%)	10,382 (42.4%)
	≥4	18,966 (4.1%)	1,231 (5.0%)
BMI during early pregnancy, kg/m^2	<18.5	9,090 (2.0%)	578 (2.4%)
	18.5-24	263,664 (57.4%)	12,539 (51.2%)
	25-30	101,561 (22.1%)	5,848 (23.9%)
	>30	46,842 (10.2%)	3,576 (14.6%)
	Unknown	38,141 (8.3%)	1,932 (7.9%)
Smoking during early pregnancy	No smoking	408,106 (88.9%)	20,110 (82.2%)
	Yes, 1-9 cig./day	21,422 (4.7%)	2,224 (9.1%)
	Yes, ≥10 cig./day	5,891 (1.3%)	867 (3.5%)

	Unknown	23,879 (5.2%)	1,272 (5.2%)
Hypertensive disorders	No	446,061 (97.1%)	23,450 (95.8%)
	Gestational hypertension/preeclampsia	4,662 (1.0%)	389 (1.6%)
	Pregestational	8,575 (1.9%)	634 (2.6%)
Diabetic disorders	No	446,824 (97.3%)	23,507 (96.1%)
	Gestational	4,896 (1.1%)	411 (1.7%)
	Pregestational	7,578 (1.6%)	555 (2.3%)
Pre-existing psychiatric disorder	No	429,671 (93.5%)	15,463 (63.2%)
	Depression	9,351 (2.0%)	4,894 (20.0%)
	Other psychiatric disorder	20,276 (4.4%)	4,116 (16.8%)
Pregnancy outcomes			
Mode of delivery	Vaginal, not assisted	358,251 (78.0%)	17,394 (71.1%)
	Vaginal, assisted	32,577 (7.1%)	1,855 (7.6%)
	Caesarean section	68,470 (14.9%)	5,224 (21.3%)
Gestational age, weeks	22-31	3,097 (0.7%)	281 (1.1%)
	32-36	18,295 (4.0%)	1,406 (5.7%)
	37-40	319,685 (69.6%)	17,513 (71.6%)
	≥41	118,221 (25.7%)	5,273 (21.5%)
Birth weight, g	<1500	2,612 (0.6%)	225 (0.9%)
	1500 to <2500	10,888 (2.4%)	882 (3.6%)
	2500 to <4200	393,096 (85.6%)	20,849 (85.2%)
	≥4200	51,905 (11.3%)	2,472 (10.1%)
	Unknown	797 (0.2%)	45 (0.2%)
Child loss	No	457,392 (99.6%)	24,252 (99.1%)
	Stillbirth	1,301 (0.3%)	154 (0.6%)
	Infant death[b]	605 (0.1%)	67 (0.3%)

Abbreviations: IQR, interquartile range; BMI, body mass index; cig., cigarette;

[a] Country of birth: *Nordic* indicated women born in Nordic countries including Denmark, Finland, Iceland, Norway, and Sweden. *Other/unknown* referred to women born in other countries or with no information (<0.01%).

[b] Infant death referred to offspring died within first year after birth.

Table S3 Hazard ratios (HRs) with 95% confidence intervals (CIs) for all-cause mortality among patients with perinatal depression,compared to matched unaffected individuals or full siblings, overall and by pre-existing psychiatric disorder, a complete case analysis.

	Population-matched cohort	Sibling cohort
**Overall association**		
**No perinatal depression**		
N of deaths (rate[a])	1,337 (0.24)	609 (0.26)
**Perinatal depression**		
N of deaths (rate[a])	422 (0.75)	98 (0.59)
HR (95% CI), model 1[b]	3.09 (2.76 to 3.45)	2.77 (1.63 to 4.69)
HR (95% CI), model 2[c]	2.72 (2.42 to 3.05)	2.60 (1.50 to 4.49)
HR (95% CI), model 3[d]	2.09 (1.83 to 2.38)	2.15 (1.16 to 3.98)
**Without pre-existing psychiatric disorder**		
**No perinatal depression**		
N of deaths (rate[a])	1,193 (0.23)	546 (0.25)
**Perinatal depression**		
N of deaths (rate[a])	208 (0.56)	47 (0.43)
HR (95% CI), model 1[b]	2.39 (2.04 to 2.79)	2.23 (1.15 to 4.34)
HR (95% CI), model 2[c]	2.21 (1.89 to 2.59)	2.12 (1.07 to 4.19)
HR (95% CI), model 3[d]	2.09 (1.79 to 2.45)	2.04 (1.00 to 4.16)
**With pre-existing psychiatric disorder**		
**No perinatal depression**		
N of deaths (rate[a])	144 (0.44)	63 (0.50)
**Perinatal depression**		
N of deaths (rate[a])	214 (1.12)	51 (0.91)
HR (95% CI), model 1[b]	2.37 (1.87 to 3.01)	2.28 (0.93 to 5.58)
HR (95% CI), model 2[c]	2.17 (1.70 to 2.76)	2.18 (0.87 to 5.45)
HR (95% CI), model 3[d]	2.09 (1.63 to 2.68)	2.07 (0.80 to 5.34)

[a] Per 1 000 person-years, unadjusted.

[b] Maternal age and calendar year at delivery (i.e., the matching factors) were inherently adjusted for in the population-matched cohort; and were controlled for in the sibling cohort.

[c] Demographic characteristics including educational level, annual household income, country of birth (for population-matched cohort) and cohabitation status were additional adjusted for.

[d] Pregnancy characteristics including BMI and smoking during early pregnancy, parity, pre-existing psychiatric disorder (for overall association), and diabetic and hypertensive disorders were additionally adjusted for.

Table S4 Hazard ratios (HRs) with 95% confidence intervals (CIs) for all-cause mortality among patients with antepartum depression,compared to matched unaffected individuals or full siblings, overall and by pre-existing psychiatric disorder.

	Population-matched cohort	Sibling cohort
**Overall association**		
**No antepartum depression**		
N of deaths (rate[a])	922 (0.26)	694 (0.26)
**Antepartum depression**		
N of deaths (rate[a])	272 (0.75)	56 (0.54)
HR (95% CI), model 1[b]	2.87 (2.50 to 3.28)	1.90 (1.03 to 3.50)
HR (95% CI), model 2[c]	2.48 (2.15 to 2.86)	1.80 (0.97 to 3.34)
HR (95% CI), model 3[d]	1.62 (1.34 to 1.94)	1.45 (0.68 to 3.08)
**Without pre to existing psychiatric disorder**		
**No antepartum depression**		
N of deaths (rate[a])	814 (0.25)	621 (0.25)
**Antepartum depression**		
N of deaths (rate[a])	112 (0.52)	21 (0.34)
HR (95% CI), model 1[b]	2.00 (1.63 to 2.45)	1.58 (0.67 to 3.76)
HR (95% CI), model 2[c]	1.87 (1.52 to 2.30)	1.58 (0.66 to 3.77)
HR (95% CI), model 3[d]	1.53 (1.21 to 1.94)	1.23 (0.45 to 3.34)
**With pre-existing psychiatric disorder**		
**No antepartum depression**		
N of deaths (rate[a])	108 (0.53)	73 (0.51)
**Antepartum depression**		
N of deaths (rate[a])	160 (1.07)	35 (0.82)
HR (95% CI), model 1[b]	2.03 (1.54 to 2.66)	1.41 (0.59 to 3.39)
HR (95% CI), model 2[c]	1.83 (1.38 to 2.42)	1.37 (0.56 to 3.36)
HR (95% CI), model 3[d]	1.81 (1.32 to 2.48)	1.58 (0.58 to 4.30)

[a] Per 1 000 person-years, unadjusted.
[b] Maternal age and calendar year at delivery (i.e., the matching factors) were inherently adjusted for in the population-matched cohort; and were controlled for in the sibling cohort.
[c] Demographic characteristics including educational level, annual household income, country of birth (for population-matched cohort) and cohabitation status were additional adjusted for.
[d] Pregnancy characteristics including BMI and smoking during early pregnancy, parity, pre-existing psychiatric disorder (for overall association), and diabetic and hypertensive disorders were additionally adjusted for.

Table S5 Hazard ratios (HRs) with 95% confidence intervals (CIs) for all-cause mortality among patients with postpartum depression,compared to matched unaffected individuals or full siblings, overall and by pre-existing psychiatric disorder.

	Population-matched cohort	Sibling cohort
**Overall association**		
**No postpartum depression**		
N of deaths (rate[a])	646 (0.24)	694 (0.26)
**Postpartum depression**		
N of deaths (rate[a])	250 (0.92)	60 (0.73)
HR (95% CI), model 1[b]	3.83 (3.31 to 4.44)	3.81 (1.88 to 7.74)
HR (95% CI), model 2[c]	3.46 (2.97 to 4.03)	3.44 (1.65 to 7.15)
HR (95% CI), model 3[d]	2.79 (2.32 to 3.34)	3.46 (1.41 to 8.49)
HR (95% CI), model 4[e]	2.71 (2.26 to 3.26)	3.87 (1.56 to 9.60)
**Without pre-existing psychiatric disorder**		
**No postpartum depression**		
N of deaths (rate[a])	572 (0.23)	621 (0.25)
**Postpartum depression**		
N of deaths (rate[a])	141 (0.69)	36 (0.58)
HR (95% CI), model 1[b]	3.10 (2.56 to 3.75)	2.50 (1.08 to 5.81)
HR (95% CI), model 2[c]	2.93 (2.41 to 3.56)	2.25 (0.95 to 5.32)
HR (95% CI), model 3[d]	2.78 (2.24 to 3.44)	2.63 (0.97 to 7.16)
HR (95% CI), model 4[e]	2.68 (2.16 to 3.34)	2.94 (1.07 to 8.07)
**With pre-existing psychiatric disorder**		
**No postpartum depression**		
N of deaths (rate[a])	74 (0.45)	73 (0.51)
**Postpartum depression**		
N of deaths (rate[a])	109 (1.62)	24 (1.18)
HR (95% CI), model 1[b]	3.18 (2.27 to 4.46)	6.08 (1.28 to 28.95)
HR (95% CI), model 2[c]	2.98 (2.11 to 4.21)	6.57 (1.31 to 32.95)
HR (95% CI), model 3[d]	2.83 (1.93 to 4.15)	9.86 (1.05 to 92.35)
HR (95% CI), model 4[e]	2.84 (1.93 to 4.18)	11.19 (1.18 to 105.80)

[a] Per 1 000 person-years, unadjusted.

[b] Maternal age and calendar year at delivery (i.e., the matching factors) were inherently adjusted for in the population-matched cohort; and were controlled for in the sibling cohort.

[c] Demographic characteristics including educational level, annual household income, country of birth (for population-matched cohort) and cohabitation status were additional adjusted for.

[d] Pregnancy characteristics including BMI and smoking during early pregnancy, parity, pre-existing psychiatric disorder (for overall association), and diabetic and hypertensive disorders were additionally adjusted for.

[e] Birth outcomes were additionally controlled for in the model, including delivery mode, gestational age, birth weight, and child loss.

Table S6 Hazard ratios (HRs) with 95% confidence intervals (CIs) for all-cause mortality among patients with perinatal depression, compared to matched unaffected individuals, by time since the index-date of perinatal depression diagnosis.

	Perinatal depression		Antepartum depression		Postpartum depression	
	No of deaths (rate[a]) in unexposed/exposed individuals	HR (95% CI) [b]	No of deaths (rate[a]) in unexposed/exposed individuals	HR (95% CI) [b]	No of deaths (rate[a]) in unexposed/exposed individuals	HR (95% CI) [b]
By time since index date for all women						
≤1 year	65 (0.10)/70 (0.82)	6.87 (4.08 to 11.56)	50 (0.11)/22 (0.46)	3.45 (1.58 to 7.55)	15 (0.08)/48 (1.25)	29.26 (9.28 to 92.23)
2-4 years	496 (0.18)/174 (0.62))	1.85 (1.47 to 2.33)	275 (0.18)/83 (0.53))	1.44 (1.02 to 2.02)	221 (0.18)/91 (0.74)	2.33 (1.70 to 3.21)
5-9 years	601 (0.31)/178 (0.89)	1.90 (1.53 to 2.36)	329 (0.30)/102 (0.91)	1.61 (1.18 to 2.20)	272 (0.32)/76 (0.87)	2.23 (1.64 to 3.03)
≥10 years	378 (0.52)/100 (1.36)	1.88 (1.39 to 2.53)	268 (0.56)/65 (1.33)	1.57 (1.08 to 2.28)	110 (0.45)/35 (1.43)	2.53 (1.48 to 4.33)
By time since index date among women with deliveries during 2001-2009						
<1 year	39 (0.16)/25 (0.76)	3.47 (1.37 to 8.81)	29 (0.16)/7 (0.39)	1.11 (0.21 to 5.84)	10 (0.15)/18 (1.21)	14.05 (1.91 to 103.20)
2-4 years	260 (0.20)/87 (0.66)	1.89 (1.36 to 2.62)	145 (0.21)/42 (0.58)	1.48 (0.90 to 2.42)	115 (0.20)/45 (0.76)	2.53 (1.58 to 4.04)
5-9 years	504 (0.33)/142 (0.90)	1.83 (1.44 to 2.33)	275 (0.33)/80 (0.92)	1.54 (1.09 to 2.19)	229 (0.33)/62 (0.88)	2.17 (1.54 to 3.06)
≥10 years	378 (0.52)/100 (1.36)	1.88 (1.39 to 2.53)	268 (0.56)/65 (1.33)	1.57 (1.08 to 2.28)	110 (0.45)/35 (1.43)	2.53 (1.48 to 4.33)
By time since index date among women with deliveries during 2010-2017						
<1 year	26 (0.06)/45 (0.85)	16.44 (7.15 to 37.81)	21 (0.07)/15 (0.51)	5.25 (1.67 to 16.52)	5 (0.04)/30(1.28)	3.67 (2.55 to 5.27)[c]
2-4 years	236 (0.17)/87 (0.59)	1.89 (1.37 to 2.62)	130 (0.16)/41 (0.49)	1.41 (0.86 to 2.31)	106 (0.17)/46 (0.72)	
5-9 years	97 (0.24)/36 (0.86)	2.37 (1.41 to 3.98)	54 (0.22)/22 (0.88)	2.02 (0.92 to 4.47)	43 (0.26)/14 (0.83)	3.03 (1.31 to 6.99)
≥10 years	-	-	-	-	-	-

[a] Per 1000 person-years, unadjusted.
[b] HRs for perinatal and antepartum depression were adjusted for educational level, annual household income, country of birth, cohabitation status, parity, smoking and BMI during early pregnancy, pre-existing psychiatric disorder, diabetic and hypertensive disorders. HRs for postpartum depression were additionally controlled for delivery mode, gestational age, birth weight, and child loss.
[c] HR was calculated during 0 to 4 years due to the small number of cases within the first year of follow-up.

Table S7 Hazard ratios (HRs) with 95% confidence intervals (CIs) for cause-specific mortality among women with antepartum and postpartum depression, compared to their matched unaffected individuals.

	**Antepartum depression**		**Postpartum depression**	
	**No of deaths (rate[a]) in unexposed/exposed individuals**	**HR (95% CI)** [b]	**No of deaths (rate[a]) in unexposed/exposed individuals**	**HR (95% CI)** [b]
Death due to unnatural cause	182 (0.05)/144 (0.39)	2.90 (2.12 to 3.96)	123 (0.05)/145 (0.53)	7.02 (5.03 to 9.80)
Suicide	65 (0.02)/70 (0.19)	5.06 (3.16 to 8.10)	52 (0.02)/78 (0.29)	12.17 (7.08 to 20.91)
Accident	76 (0.02)/43 (0.12)	1.91 (1.06 to 3.45)	45 (0.02)/35 (0.13)	6.07 (3.16 to 11.67)
Others	41 (0.01)/31 (0.08)	3.16 (1.30 to 7.70)	26 (0.01)/32 (0.12)	14.33 (4.42 to 46.46)
Death due to natural cause	740 (0.21)/128 (0.34)	1.13 (0.88 to 1.45)	523 (0.19)/104 (0.38)	1.64 (1.28 to 2.11)
Cancer	546 (0.16)/66 (0.18)	0.93 (0.68 to 1.28)	381 (0.14)/72 (0.26)	1.80 (1.35 to 2.41)
- excluding women with a history of cancer	512 (0.15)/62 (0.17)	0.91 (0.66 to 1.27)	310 (0.12)/35 (0.13)	1.13 (0.77 to 1.66)
Cardiovascular disease	77 (0.02)/19 (0.05)	1.63 (0.75 to 3.55)	57 (0.02)/11 (0.04)	2.12 (0.77 to 5.84)
- excluding women with a history of cardiovascular disease	66 (0.02)/16 (0.04)	1.72 (0.74 to 3.98)	47 (0.02)/10 (0.04)	2.33 (0.79 to 6.91)
Others	117 (0.03)/43 (0.12)	1.97 (1.17 to 3.33)	85 (0.03)/21(0.08)	1.29 (0.64 to 2.59)

[a] Per 1 000 person-years, unadjusted.[b] HRs for perinatal and antepartum depression were adjusted for educational level, annual household income, country of birth, cohabitation status, parity, smoking and BMI during early pregnancy, pre-existing psychiatric disorder, diabetic and hypertensive disorders. HRs for postpartum depression were additionally controlled for delivery mode, gestational age, birth weight, and child loss.

Table S8 The most common causes of death due to cancer among women with perinatal depression.

Perinatal depression			Antepartum depression			Postpartum depression		
**ICD 10**	**N**	**Rate [a]**	**ICD 10**	**N**	**Rate [a]**	**ICD 10**	**N**	**Rate[a]**
C50 Malignant neoplasm of breast	36	0.06	C50 Malignant neoplasm of breast	16	0.04	C50 Malignant neoplasm of breast	20	0.07
C53 Malignant neoplasm of cervix uteri	17	0.03	C53 Malignant neoplasm of cervix uteri	8	0.02	C53 Malignant neoplasm of cervix uteri	9	0.03
C34 Malignant neoplasm of bronchus and lung	9	0.01	C43 Malignant melanoma of skin	5	0.01	C34 Malignant neoplasm of bronchus and lung	6	0.02
C71 Malignant neoplasm of brain	9	0.01	C71 Malignant neoplasm of brain	5	0.01	C18 Malignant neoplasm of colon	4	0.01
C18 Malignant neoplasm of colon	8	0.01	C18 Malignant neoplasm of colon	4	0.01	C71 Malignant neoplasm of brain	4	0.01

[a] Per 1 000 person-years, unadjusted.

Table S9 Hazard ratios (HRs) with 95% confidence intervals (CIs) for cause-specific mortality among women with perinatal depression and by pre-existing psychiatric disorder, compared to their matched unaffected individuals, by common cause of death, with additional adjustment for pre-existing comorbidities.

	Overall	Women without pre-existing psychiatric disorder	Women with pre-existing psychiatric disorder
	HR (95% CI) [a]	HR (95% CI) [a]	HR (95% CI) [a]
**Death due to unnatural cause**	4.29 (3.45 to 5.34)	4.86 (3.68 to 6.44)	3.43 (2.31 to 5.09)
Suicide	6.35 (4.62 to 8.71)	7.87 (5.25 to 11.79)	4.16 (2.29 to 7.56)
Accident	3.14 (2.08 to 4.72)	2.89 (1.70 to 4.93)	3.58 (1.70 to 7.51)
Others	5.83 (3.15 to 10.81)	7.37 (3.42 to 15.89)	3.95 (1.46 to 10.65)
**Death due to natural cause**	1.48 (1.21 to 1.80)	1.50 (1.19 to 1.89)	1.36 (0.91 to 2.02)
Cancer	1.49 (1.16 to 1.91)	1.63 (1.24 to 2.15)	1.00 (0.56 to 1.77)
- excluding women with a history of cancer	1.06 (0.78 to 1.43)	1.11 (0.79 to 1.56)	0.93 (0.49 to 1.75)
Cardiovascular disease	1.75 (0.95 to 3.20)	1.22 (0.56 to 2.68)	4.13 (1.16 to 14.65)
- excluding women with a history of cardiovascular disease	1.87 (0.97 to 3.59)	1.53 (0.68 to 3.43)	2.97 (0.78 to 11.28)
Others	1.57 (1.03 to 2.38)	1.59 (0.95 to 2.68)	1.28 (0.63 to 2.60)

[a] HRs for perinatal and antepartum depression were adjusted for educational level, annual household income, country of birth, cohabitation status, parity, smoking and BMI during early pregnancy, pre-existing psychiatric disorder (for overall association), diabetic and hypertensive disorders, as well as previous comorbidities using Charlson Comorbidity Index.

Table S10 Hazard ratios (HRs) with 95% confidence intervals (CIs) for all-cause mortality among patients with perinatal depression, compared to matched unaffected individuals or unaffected full siblings, by different ascertainments of perinatal depression.

Model information	Population-based matched cohort		Sibling cohort	
	No of deaths (mortality[a]) in unexposed/exposed individuals	HR (95% CI)	No of deaths (mortality[a]) in unexposed/exposed individuals	HR (95% CI)
**Restricting to perinatal depression ascertained from clinical diagnoses**				
Perinatal depression [b]	387 (0.25)/148 (0.92)	2.34 (1.79 to 3.06)	694 (0.26)/36 (0.80)	2.12 (0.77 to 5.80)
Antepartum depression [b]	234 (0.25)/75 (0.77)	1.60 (1.07 to 2.40)	694 (0.26)/15 (0.56)	1.39 (0.39 to 4.96)
Postpartum depression [b]	153 (0.24)/73 (1.15)	3.35 (2.32 to 4.84)	694 (0.26)/21 (1.16)	4.76 (0.89 to 25.54)
**Restricting to perinatal depression ascertained from prescriptions**				
Perinatal depression [b]	1181 (0.26)/374 (0.79)	2.11 (1.82 to 2.45)	694 (0.26)/80 (0.56)	2.23 (1.10 to 4.53)
Antepartum depression [b]	688 (0.27)/197 (0.74)	1.69 (1.37 to 2.08)	694 (0.26)/41 (0.53)	1.59 (0.65 to 3.89)
Postpartum depression [b]	493 (0.24)/177 (0.85)	2.61 (2.11 to 3.23)	694 (0.26)/39 (0.60)	3.68 (1.26 to 10.77)

[a] Per 1000 person-years, unadjusted.

[b] Maternal age and calendar year at delivery (i.e., the matching factors) were inherently adjusted for in the population- matched cohort; and were controlled for in the sibling cohort. HRs were adjusted for educational level, annual household income, maternal country of birth (for population-matched cohort), cohabitation status, parity, smoking and BMI during early pregnancy, pre-existing psychiatric disorder, diabetic and hypertensive disorders. HRs for postpartum depression were additionally controlled for delivery mode, gestational age, birth weight, and child loss.

Table S11 Hazard ratios (HRs) with 95% confidence intervals (CIs) for all-cause mortality among patients with antepartum and postpartum depression, compared to matched unaffected individuals, by time since pregnancy or delivery.

	No of deaths (mortality[a]) in unexposed/exposed individuals	Model 1[b] HR (95% CIs)	Model 2 [c] HR (95% CIs)	Model 3 [d] HR (95% CIs)	Model 4 [e] HR (95% CIs)
**Antepartum depression**					
Within 13 weeks	386 (0.23)/154 (0.86)	3.80 (3.15 to 4.59)	2.99 (2.44 to 3.66)	1.78 (1.35 to 2.34)	-
14 weeks onwards	536 (0.30)/118 (0.64)	2.18 (1.78 to 2.66)	2.05 (1.67 to 2.52)	1.43 (1.11 to 1.86)	-
**Postpartum depression**					
Within 6 months	297 (0.24)/109 (0.85)	3.60 (2.88 to 4.49)	3.25 (2.59 to 4.08)	2.75 (2.09 to 3.61)	2.68 (2.03 to 3.53)
7-12 months	349 (0.24)/141 (0.98)	4.03 (3.31 to 4.92)	3.63 (2.96 to 4.46)	2.84 (2.23 to 3.63)	2.76 (2.16 to 3.54)

[a] Per 1000 person-years, unadjusted.

[b] Maternal age and calendar year at delivery (i.e., the matching factors) were inherently adjusted for in the population- matched cohort.

[c] Demographic characteristics including educational level, annual household income, country of birth and cohabitation status were additional adjusted for.

[d] Pregnancy characteristics including BMI and smoking during early pregnancy, parity, pre-existing psychiatric disorder, and diabetic and hypertensive disorders were additionally adjusted for.

[e] Pregnancy outcomes including delivery mode, gestational age, birth weight, and child loss were further adjusted for.

Supplementary material 2 –Healthcare services for mother and child in Sweden

In Sweden, most pregnant women attend maternal healthcare centers. At enrollment, a risk assessment of the pregnancy, including psychiatric anamnesis, is conducted by the midwife [1]. Pregnant women identified at risk for PND should be recommended to a medical doctor's visit/consultation/planning round in early pregnancy for care planning of pregnancy and childbirth [2]. In case of social problems and/or unhealthy lifestyles, extra support measures are planned via the midwife at the maternal healthcare centers. After delivery, 99.0% children are enrolled to child health services, and focus are shifted to the born baby. Since 2010, it is stated in the national guidelines that at approximately 8 weeks postpartum, midwifes at the child health services should provide mothers with a screening for postpartum depression. If a woman is screened positive, the woman should be referred to a medical doctor in primary care, a psychologist, or a psychiatrist [3]. The medical doctor/psychologist/psychiatrist assess depression using Diagnostic and Statistical Manual of mental disorders (DSM) criteria. All diagnoses entered to patient records by doctors at the specialized outpatient care within the Swedish healthcare are registered in the National Patient Register [4]. Information registered by healthcare providers in patient records during visits to maternal healthcare centers and maternity care for pregnancies resulting in a live birth, are registered in the Medical Birth Register [5]. After diagnosis, the doctor/psychologist/psychiatrist decides on appropriate treatment method, including counselling or medical treatment, where SSRIs are the most prescribed medications. In Sweden, antidepressants are prescribed by medical doctors or psychiatrists. The National Prescribed Drug Register [6] contains information on all medications that are dispensed through a prescription at any pharmacy in Sweden since July 2005.

References:

1 Region Stockholm. Riskbedömning 2022. Kunskapsstöd För Vårdgivare. https://kunskapsstodforvardgivare.se/download/18.534008331800299c7a9ca14/1650452296108

2 Region Stockholm. Basprogram. Kunskapsstöd För Vårdgivare. https://kunskapsstodforvardgivare.se/omraden/kvinnosjukdomar-och-forlossning/riktlinjer-for-bmm/barnmorskemottagning/graviditet/riktlinjer/basprogram

3 Wickberg B. Screening med EPDS för depression hos nyblivna mammor. Rikshandboken Barnhälsovård. https://www.rikshandboken-bhv.se/metoder--riktlinjer/screening-med-epds/

4 National Patient Register. Socialstyrelsen. https://www.socialstyrelsen.se/en/statistics-and-data/registers/national-patient-register/ (accessed 27 Oct 2022).

5 National Medical Birth Register. Socialstyrelsen. https://www.socialstyrelsen.se/en/statistics-and-data/registers/national-medical-birth-register/ (accessed 27 Oct 2022).

6 National Prescribed Drug Register. Socialstyrelsen. https://www.socialstyrelsen.se/en/statistics-and-data/registers/national-prescribed-drug-register/ (accessed 27 Oct 2022).
